# Recent advances in nanomaterial-based drug delivery systems for melanoma therapy

**DOI:** 10.5599/admet.2088

**Published:** 2023-10-24

**Authors:** Rabinarayan Parhi, Partha Pratim Kaishap, Goutam Kumar Jena

**Affiliations:** 1 Department of Pharmaceutical Sciences, Susruta School of Medical and Paramedical Sciences, Assam University (A Central University), Silchar-788011, Assam, India; 2 Roland Institute of Pharmaceutical Sciences, Berhampur-7600010, Odisha, India

**Keywords:** cancer, nanoparticles, carbon nanotubes, genetic mutation, radiation therapy, targeted therapy

## Abstract

**Background and Purpose:**

Safe and effective drug delivery is crucial for the treatment of cancer, which is quite impossible to achieve through traditional methods. Among all types of cancer, skin melanoma is known for its aggressive metastasizing ability and an unprecedented higher degree of lethalness, limiting the overall therapeutic efficacy. Here, we focus on the different types of nanomaterials (NMs) and their drug delivery applications against melanoma.

**Experimental Approach:**

All relevant publications, including research papers, reviews, chapters and patents, were assessed using search engines such as Scopus and PubMed, up to the end of August of 2023. The keywords used in the search were: nanomaterials, melanoma, drug delivery routes for melanoma, and nanomaterial-based drug delivery systems (DDS). Most of the publications out of 234 cited in this review are from the last five years.

**Key Results:**

The recent advancement and mechanism of action of various NMs against melanoma, including inorganic metallic and carbon-based NMs, organic polymeric and lipid-based NMs, and cell-derived vesicles are discussed. We also focus on the application of different NMs in the delivery of therapeutic agents for melanoma therapy. In addition, the skin and melanoma, genetic mutation and pathways for melanoma, conventional treatment options, and delivery routes for therapeutic agents are also discussed briefly.

**Conclusion:**

There are few NM-based DDS developed in the lab set up recently. The findings of this review will pave the path for the development of NM-based DDS on an industrial scale and help in the better management of skin melanoma.

## Introduction

Cancer is one of the most dreadful diseases with a serious impact worldwide. It is characterised by uncontrolled growth and proliferation of cells and is mainly related to changing nutrition and lifestyle [1]. This is clearly witnessed from a report published by the National Cancer Institute (NCI) that in 2016, about 1,700,000 new cancer cases were diagnosed in the United States, and it caused the death of 595,690 people [2]. The high death rate is mainly because of poor prognosis for many cancer types. The major factors contributing to cancers are carcinogens, which have the potential to cause dysplastic, hyperplastic or degenerative alternation in cell haemostasis, external factors, such as radiation, pollution and internal factors, including oxidative stress [3,4]. Cancers are classified either by their location, such as liver, brain, lung, breast, oral, skin, intestinal, *etc.*, or by their histological characteristics, including sarcoma on connective and supportive tissues, melanoma on the skin, carcinoma on epithelial tissues, myeloma on bone marrow, lymphoma on lymphatic systems *etc.* [5].

Melanoma is a highly aggressive form of skin cancer and a prime global health concern due to its metastasizing nature and higher mortality rate. There is a huge geographical variation in the occurrence across world regions and countries. As per the most recent epidemiological assessments of global cancer data, the estimated number of new melanoma cases is 325,000, and deaths due to melanoma reached 57,000 in 2020 [6]. If this rate continues, the global burden from melanoma is estimated to reach a staggering 510,000 new cases and 96,000 deaths by 2040 [7]. The most concerning aspect of melanoma is that there is a significant death of individuals aged between 20-35 years [8]. The most prominent factors for melanoma are fair skin complexation, family history of the disease, appearance of moles in the skin, immunosuppression and excessive exposure to ultraviolet (UV) radiation [7]. The in-depth study on the immunology of melanoma suggested that the involvement of mutation in the genes such as v-Raf murine sarcoma viral oncogene homolog B1 (BRAF), neuroblastoma RAS viral oncogene homolog (NRAS) and nuclear factor I (NFI), called as triple-wild type genotype, complicated the treatment process [9,10]. Melanoma has a 5-year survival rate (93 %) and is even curable by surgical resection if diagnosed at early stages. If not detected in the early stages (stage I or II), melanoma metastasizes very fast, with a survival rate of only six months and a decrease of 81 % in the 5-year survival rate [11]. However, the early-stage detection of tumours is challenging due to the absence of clinical symptoms until the disease reaches the metastatic stage, the lack of public awareness and the unavailability of tumour-detection markers [12].

Currently, five types of standard treatment options are available for patients suffering from melanoma, including surgery, chemotherapy, immunotherapy, targeted therapy and radiation therapy [13]. Among them, commonly used treatment options are surgery (limited to early stage), chemotherapy using anticancer therapeutic agents such as dacarbazine and radiotherapy using radiation are limited by serious systemic effect and therapeutic ineffectiveness due to non-selectivity, large skin defects due to radiation, high risk of reoccurrence and therapeutic resistance [14,15]. Then comes FDA-approved systemic immunotherapy employing cytotoxic T-lymphocyte–associated antigen 4 (CTLA-4) and programmed death 1 (PD-1) immune checkpoint inhibitors. To avoid systemic side effects, targeted therapy was introduced where BRAF inhibitors (*e.g.*, vemurafenib), MEK inhibitors (*e.g.*, trametinib) and c-KIT inhibitors specifically target the affected mutated melanoma cells [16,17]. These treatment options significantly showed progression-free survival and overall longevity of the patients suffering from malignancy. However, they suffer due to a lack of efficacy, instances of adverse effects and, more importantly, resistance [18]. More recently, alternative therapy has been developed, such as photodynamic therapy (PDT) [19] and photothermal therapy (PTT) [20]. However, the therapeutic efficacies are majorly dependent on the specific accumulation of therapeutic agents involved, such as photosensitizer in the case of PDT and photothermal agents in the case of PTT at the melanoma sites [21]. Therefore, an urgent need is to devise new effective therapy to treat metastatic melanoma effectively.

In this context, nanotechnology involving nanoparticles (NPs) of dimension generally less than 100 nm holds a crucial potential as an alternative therapy for melanoma due to their target-specific delivery of therapeutic agents, low side effects, increased circulation time, higher bioavailability, sustained drug release and ability to carry and delivery multiple therapeutic agents for synergistic anticancer effect [22,23]. In addition, they potentially enhance the solubility of poorly soluble drugs, protect the encapsulated drugs from surrounding challenging environments from degradation, and improve intracellular and cross-biological membrane delivery [24]. Based on drug delivery applications, nanomaterials (NMs) are mainly classified into two categories: (i) inorganic NMs such as silver, platinum, gold, iron, copper etc, (metallic NMs), graphene and its derivatives (carbon-based NMs), (ii) organic NMs including the NPs derived from polymer (polymeric nanocapsules, nanospheres, dendrimers, nanogel, etc) and lipids (liposomes, niosomes, micelles, solid lipid nanoparticles (SLN), *etc.*) [25,26]. These NPs can carry and deliver therapeutic molecules such as chemotherapeutic agents, radiotherapeutics, photosensitizers, photothermal agents, monoclonal antibodies (mAb), peptides and proteins to the target site. Additionally, inorganic NPs can directly kill melanoma cells through DNA damage, oxidative stress and cell membrane damage [27].

## Skin cancer and melanoma

**Figure 1.**
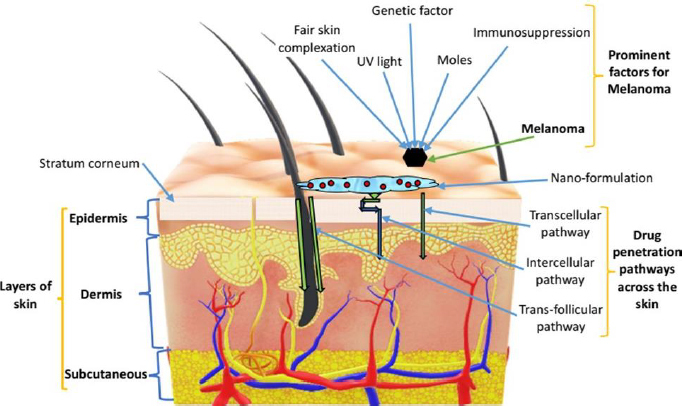
Diagrammatic representation of different skin layers, prominent factors of melanoma and the skin penetration pathways

Skin is the largest organ of the human body that performs several functions, including the protection of internal body parts from pathogens and exogenous particles and homeostasis of the internal medium among the important ones [28,29]. Histologically, the skin comprises three distinct layers: the superficial epidermis, middle dermis and lower hypodermis or subcutaneous. The skin also holds many types of cells, such as melanocytes, keratinocytes, Langerhans, and dendritic cells in the epidermis. **Figure 1** represents different skin layers along with prominent factors of melanoma and the skin penetration pathways.

There are two subtypes of cancers such as melanoma and non-melanoma, most often diagnosed in the skin of the Caucasian population. Non-melanoma subtypes of skin cancer are most prevalent and affect mainly keratinocytes or epidermal cells. It includes basal cell carcinoma, sebaceous cell carcinoma, squamous cell carcinoma, Bowen’s disease, Adnexal tumours, Merkel cell carcinoma and Kaposi sarcoma [30]. However, melanoma skin cancer is confined to melanocytes and is considered as most aggressive and fatal [31].

Melanoma is a malignant transformation of pigment-producing melanocytes mainly present in the stratum basale of the epidermal layer of skin, inner ear, uvea of eyes, bones and hearts. Various epidemiological studies have proved that the main predisposing factors for melanoma include excessive UV light exposure, a family history of melanoma, moles and a poor immune system [32]. There is a higher risk of melanoma in the population with pale skin colour, *i.e.*, Caucasians, than in the population with dark skin, *i.e.*, African and East Asians [33]. The pigment melanin in the skin is responsible for “suntan” and has the function of protecting the skin against harmful UV radiation from the sun [34]. However, excessive sun exposure may have a higher risk for melanoma. The origin and progress of melanoma is a multistep process with five distinct stages (stage 0 to stage 4) bearing histological and clinical characteristics [12,35,36].

Stage 0: It is also referred to as melanoma in situ with the presence of abnormal melanocytes on the upper layer of the epidermis. Nevi are benign skin lesions, but most often, they transform into malignant melanoma. Increased melanoma proliferation may lead to these acquired nevi (mole) without dysplasia.

Stage 1: Abnormal nevi develops into dysplastic nevi of size ranges between 1 to 2mm, showing abnormal differentiation.

Stage 2: The dysplastic nevi continue to differentiate and enter into the radial growth phase (RGP) primary tumour with a size of approximately 2 to 4 mm, however, RGP is only confined to the epidermis without the ability to invade the dermis.

Stage 3: Through genetic alteration, RGP acquired invasive potential and started to infiltrate the dermis and spread to a number of lymph nodes. This is called the vertical growth phase (VGP) and restricts the treatment option due to their self-sufficient growth signals and invading ability.

Stage 4: Metastatic lesson is developed and spread to distant vital organs such as the liver, brain, lung and gastrointestinal tract (GIT).

Melanoma is basically classified into four types: superficial spreading melanoma, nodular melanoma, lentiginous melanoma and acral lentiginous melanoma [37]. The superficial spreading melanoma accounts for 70 % of total melanoma and originates from pre-existing moles. It is frequently seen on the backs of males and the legs of women and can be treated with appropriate excision [38]. The nodular form of melanoma is the most aggressive form and grows faster without RGP. They originate from pre-existing bumps with blue-black to purplish colour, accounting for 15-30 % of total melanoma manifested. Lentiginous melanoma is generally observed as a large and flat lesion on the face of light-skinned older patients with sun exposure for a long time and accounts for 4-15 % of total melanoma incidence [39]. Acral lentiginous melanoma lesions account for only 2-8 % of melanomas and are observed in the palms and soles [38].

## Genetic mutation and pathways for melanoma

The malignant transformation of melanocytes mainly involves genetic alteration resulting in oncogenic mutations such as BRAF (B-Raf proto-oncogene, serine/threonine kinase) mutation, the appearance of cyclin-dependent kinase inhibitor 2A (CDKN2A) locus, and mutations in melanin related genes such as melanocortin-1 receptor, tyrosinase, *etc.* [40]. The potential cause for these mutations is the enrichment of cytosine to thymine or guanine to adenine induced by UV radiation [41]. The BRAF oncogene encodes for protein kinase in the mitogen-activated protein kinase (MAPK) pathway (RTK-RAS-RAF-MEK-ERK) [42]. Mutation in the BRAF and NRAS accounts for 50 and 30 % of the total cause of melanoma, respectively. Another important signalling pathway involved in cell growth and apoptosis in melanoma is the activation of the phosphatidylinositol 3-kinase (P13K/AKT) pathway. Tyrosine kinase receptors directly activate the PI3 kinases, resulting in the phosphorylation of PIP2 to PIP3. These events led to AKT’s phosphorylation, which induces effector molecules for regulating cell proliferation. Activated AKT inhibits GSK3β and induces the accumulation of β-catenin and their movement into the nucleus. Further, β-catenin coactivates transcription factor (T-cell factor), leading to the upregulation of c-MYC and cyclin D and inducing cell proliferation. In addition, mutations in tyrosine kinase receptor (c-KIT) activate the PI3K/AKT pathways and induce tumour progression [43].

The significant advancement in immunology indicated that melanin tumorigenesis is not only caused due to genetic mutation but also induced by immunology. This is justified because:

- melanoma can disrupt antigen presentation mechanism by avoiding immune recognition and elimination. For example, downregulation of major histocompatibility complex class-I molecules, thereby putting an end to antigen processing reaction.
- upregulation involving programmed death ligand-1 (PD-L1) expression on the melanoma surface resulting in the suppression of the effector function of tumour infiltration lymphocytes *via* interaction between PD-1 and PD-L1 [44],
- the creation of suppressive tumour microenvironment due to the role of immunosuppressive cells in melanin transformation, including regulatory T cells (Tregs), myeloid-derived suppressor cells (MDSCs) and the enzymes and molecules released by these cells such as reactive oxygen species (ROS), arginase-1, *etc.* [45].

## Conventional treatment options and their limitations

Melanoma has a very high metastatic potential accompanied by a sudden increase in angiogenic factors. Therefore, the prognosis of melanoma is based on the stage of its progression. The standard treatment options available are (i) surgical resection, (ii) chemotherapy (using chemotherapeutic agents), (iii) Targeted therapy (oncogene targeting via melanoma signalling pathway inhibitors), (iv) Immunotherapy (immune checkpoint inhibitors), (v) radiation therapy (vi) PDT and (vii) PTT.

- **Surgical resection**: This option is adopted for patients with stage I and stage II melanoma lesions. The 5-year survival rate for surgical resection is 95 and 80 % with stage I and stage II melanoma, respectively [46]. Generally, Moh’s surgery is employed to excise layers of skin until there is no sign of a tumour, but in some instances, lymphadenectomy or amputation is essential [12,47]. However, surgical resection is not a feasible option for melanoma after metastasis into distant organs (stage IV) as the median survival of only 8-9 months and the 3-year overall survival (OS) rate is lower than 15 % [48]. Thus, in the case of non-respectable melanoma/metastatic melanoma, other treatment options are being widely used.
- **Chemotherapy**: Treatment of melanoma chemotherapeutic agents involves the use of FDA-approved dacarbazine, high-dose of interleukin-2 (IL-2) and interferon α-2b (IFN-a-2b) [29,40]. In addition, some natural bioactive compounds such as taxanes (taxol, docetaxel) and vinca alkaloids (vincristine and vinblastine) are also being considered for melanoma treatment. However, the overall objective response rates were 20, 16 and 15 % for dacarbazine, high-dose of IL-2 and IFN-a-2b), respectively [49]. Furthermore, the non-selective killing of cells involving the killing of healthy cells takes place and the resistance to the treatment with a median response time between 4-6 months has been developed by melanoma [50].
- **Targeted therapy**: Recently, the treatment of metastatic melanoma has been majorly focussed on underlying genetic mutation and immunological factors. The BRAF inhibitors (functioning through MAPK signalling pathway and inhibiting BRAF protein synthesis) such as vemurafenib dabrafenib and encorafenib, MEK inhibitors such as trametinib, binimetinib and cobimetinib and c-KIT inhibitors (*e.g.*, imatinib, nilotinib) showed initial superior response than that of dacarbazine and improvement on the progression-free survival and OS. However, BRAF inhibitor sufferers have a high relapse rate due to resistance after 6 months and show adverse effects [40,51,52].
- **Immunotherapy** involves the increase in the immune system to actively or passively fight against melanoma [47]. It involves immune checkpoint inhibitors such as ipilimumab, and inhibition of an anti-CTLA-4 mAb such as atezolizumab, avelumab, ipilimumab and anti-PD-1 such as nivolumab and pembrolizumab antagonising the activity of PD-1 demonstrated more durable responses with only a small portion of patients achieve an objective response to the treatment. In addition, the application of immune checkpoint inhibitors is limited by different immuno-related adverse effects such as nephritis, colitis, hepatitis, cardiotoxicity, vitiligo, pruritis, *etc.* [53,54]. Furthermore, immune-related adverse effects with the blockade of PD-1 limit their clinical significance [55,56].
- **Radiation therapy** involves the application of high-energy electromagnetic rays such as x- and -rays for the treatment of melanoma when spread to distal organs such as the brain, bones, etc. Radiation selectively causes neoplastic cell killing and DNA damage of the malignant cells that prevent these cells from multiplication [57].
- **PDT**: This method of melanoma treatment employs light, photosensitizer and oxygen to generate reactive oxygen species (ROS), such as singlet oxygen, against melanoma cells [58,59]. Here, the light is absorbed by photosensitizer and the light energy is stored in the singlet or triplet state of the sensitizer molecules. In the singlet state, the energy is converted into heat or emitted as light, while the triplet state generates ROS for the successful PDT. These ROS cause apoptosis and necrosis of cancer cells. PDT is a non-invasive and inexpensive method that requires proper coordination between light sources such as lasers, light-emitting diodes (LEDs) and photosensitizers such as inorganic NMs such as gold, platinum, silver, *etc*.
- **PTT** is based on the conversion of photon energy into heat source by the use of photosensitizing molecules to cause hyperthermia and subsequent selective killing of melanoma cells due to heat [60]. Thus, PTT is a hyperthermic treatment that depends on the ability of photosensitizing molecules (*e.g.*, inorganic NMs) to absorb laser irradiation and convert them into heat [61]. PTT is a widely used method in association with other therapies such as chemotherapy, immunotherapy and even PDT for better success in the treatment of melanoma [60].

Despite all the conventional treatment options, there is inevitably low efficacy and adverse effects due to the non-specific delivery of therapeutic agents to tumour cells. Therefore, nano-delivery systems involving NPs, capable of carrying therapeutic agents, hold great potential to improve drug delivery efficiency and minimise side effects. These nano-dimensional particles showed many advantages compared to conventional drug delivery, including (i) improvement of the solubility of poorly water-soluble drugs by encapsulating them, thereby increasing the bioavailability, (ii) protection of encapsulated drugs from the external environment involving the presence of enzymes which is having the potential to degrade them, (iii) surface modification through PEGylation or conjugation with suitable ligands can improve circulation time in systemic circulation and higher accumulation of therapeutic agents at tumour sites, and (iv) allow the incorporation of more than one drugs or encapsulation of more than one agent for combination therapy and derive synergistic effect for better treatment of melanoma [40].

## Delivery routes for therapeutic agents

After various treatment options for melanoma, the next answer to find out is their routes of delivery. Basically, there are three routes of delivery: topical, transdermal and parenteral routes. In most skin cancer cases, topical delivery of therapeutics is preferred because they avoid the potential toxic effects of systemic agents. It is highly desirable in pre-cancerous lesions or in-transit disease [62]. However, the topical route suffers due to the risk of metastasis and presently, there is no FDA-approved topical therapy for melanoma. In addition, the barrier nature of the stratum corneum of the epidermis prevents the entry of therapeutic agents into deeper skin layers. In this context, the transdermal route using chemical and various physical techniques remains a highly efficient route of choice in melanoma treatment. The barrier nature of stratum corneum can be reversibly altered with chemical penetration enhancers and allow the penetration of considerably larger particles compared to the topical route. NP strategy employing polymeric NPs, liposomes, niosomes, transferosomes, etc., can be seen as an alternative way to enhance the penetration of therapeutics across the epidermis [43]. If further enhancement of penetration of therapeutics is desired, then the combination of microneedle (MNs)-based minimally-invasive physical techniques can be employed alone or in combination with NMs. MN technique comprised of a number of micron-sized needles upon their application on the skin surface can penetrate the stratum corneum and thus create micro-conduits in the skin through which therapeutic molecules or NMs can pass and reach viable epidermis [63]. Due to recent success, MN techniques are combined with other therapies such as PDT, PTT, chemotherapy, and immunotherapy for synergistic effects against melanoma [64]. The main advantage of the transdermal route is the local delivery of therapeutic agents and, thereby, avoidance of systemic toxicity. The parenteral route is opted for only advanced metastatic melanoma, where other routes are ineffective. The therapeutic agents administered through the parenteral route include immunotherapeutic agents, chemotherapeutic agents and the inhibitors used in targeted therapy [65]. However, the parenteral route is limited by the non-specific delivery of therapeutic agents, leading to a reduction in efficacy, cutaneous and extracutaneous adverse effects shown by many therapeutic agents and minimum uptake of therapeutic agents in melanocytes [66].

## Classification of nanomaterials

**Figure 2.**
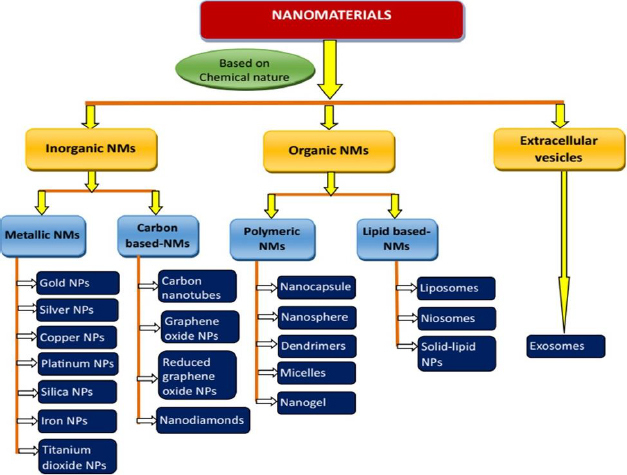
Schematic representation of classification of nanomaterials employed in the treatment of melanoma.

NMs can be classified into various groups based on different criteria such as chemical nature, dimensions, state and morphology. However, NMs employed in drug delivery applications are broadly classified into two categories: inorganic and organic NMs. Based on their chemical nature, they are classified into inorganic, organic and cell-derived/extracellular vesicles Figure 2 [67-69].

### Inorganic nanomaterials

**Figure 3.**
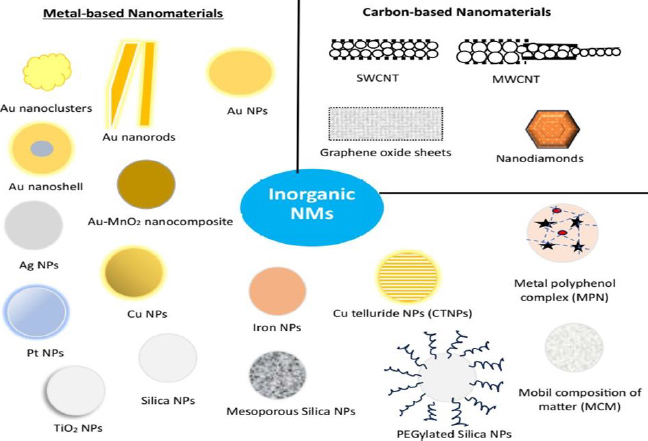
Illustration of types of inorganic NMs: metal-based NMs and carbon-based NMs. NPs: nanoparticles, CTNPs: copper telluride nanoparticles, MPN: metal polyphenol complex, MCN: mobil composition of matter, SWCNT: single-walled carbon nanotubes, MWCNT: multiwalled carbon nanotubes

Inorganic NMs have been extensively explored for decades for applications in diverse fields, including diagnostic and therapeutic applications. This is mostly due to their uniform sizes with high dispersion. In addition, inorganic NMs are comparatively facile to construct and provide high yield [70]. These NMs allows various chemical modulation on their surface, such as ligand conjugation, charge modulation, polyethylene glycol conjugation (PEGylation), and the inclusion of small-molecule probes and stimuli-responsive moieties for the improvement of drug efficiency [71,72]. Further, appropriate modification may lead to the enhancement of their water solubility and, subsequently, bioavailability. Moreover, drug molecules can be conjugated with inorganic NMs, facilitating drug delivery applications. Inorganic NMs are broadly classified into metallic NMs and carbon-based NMs (Figure 3).

#### Metal-based nanomaterials

Metals and, more particularly, their nano species have shown tremendous potential in medical research and drug delivery due to their natural availability and can be easily processed into their nanostructures. Among all, metal and their oxides in the form of NPs have great potential not only in the treatment of diseases but also in carrying drug molecules for targeted drug delivery in the case of specific diseases such as cancers. Out of various metals, gold, silver, copper, platinum, silica, titanium dioxide (TiO_2_) and iron-based NMs are used in the treatment of melanoma and are discussed below.

##### Gold nanoparticles

In 1857, Michael Faraday first reported gold NPs, producing colloidal gold through the reduction reaction of gold salts [73]. However, the therapeutic application of gold in the treatment of rheumatoid arthritis was performed in 1927 [74], followed by the use of nanogold against various rheumatoid diseases. Gold NPs have been explored in the treatment of various diseases due to (i) precise control over their particle size distribution, (ii) allowing the modification of shapes to produce cubes, nanoclusters, nanorods, spheres, nanoprisms, hexagonal platelets, etc., (iii) biocompatible inside the mammalian cell as they are inactive and not potentially separate into particles [75], (iv) surface modification through conjugation with therapeutic molecules, molecular probe, antibodies, various polymers, etc. [76], and (v) extraordinary colloidal stability and tuneable optical characteristics. In cancer treatment, high tissue permeability, biocompatibility and bioconjugation play crucial roles. The positive charge on the surface of gold NPs facilitates their internalization by the cell membrane, which is negatively charged due to the presence of lipids. In addition, they are steady against oxidation under physiological conditions such as temperature, pH conditions and ionic strength [77,78]. They can be selectively transported and precisely targeted using both active and passive targeting approaches. The accumulation of gold NPs in the tumour tissue is due to the enhanced permeability and retention (EPR) effect (the mechanism due to which high-molecular weight non-targeted drugs and prodrugs accumulated in the tissues that exhibited increased vascular permeability) and subsequently, taken up by cells through the endocytosis process [79].

Gold NMs have been utilized in various forms, such as NPs, nanorods, nanoclusters, nanoshells, nanocomposites, nanocages and so on for drug delivery in the treatment of melanoma. A few important and recent studies regarding the use of gold NMs as carriers to deliver drugs to melanoma sites are presented below. Gold nanoclusters have great potential for application in the biomedical field due to their promising optical properties. Based on this, Latorre *et al.* [80] fabricated albumin-stabilized gold nanoclusters (albumin-based nanostructures) loaded with AZD8055, a potent, selective ATP-competitive mammalian target of rapamycin (mTOR) kinase inhibitor, for its specific delivery into Uveal melanoma. In this, the nanostructure having low toxicity and exceptional stability in vivo is conjugated with the drug employing a disulfide moiety-containing linker. As a result, the drug was released selectively and effectively in the tumoral cells in the presence of a higher concentration of glutathione. The robust nanostructure induced significant cellular toxicity without affecting non-tumoral keratinocytes. Further, these nanostructures demonstrated excellent in vivo activity by decreasing the tumour surface compared to the free drug in the mice model. These nanostructures have the capability of providing the same result, employing 23 times lower doses than those employed in the previous study.

In one study, biocompatible gold NPs were biosynthesized with an aqueous extract of *Siberian ginseng* (SG-GNPs) in a cost-effective manner. The biological ingredients in *Siberian ginseng* act as a reducing agent, reducing gold ions to gold NPs. The active ingredient chalcones present in *Siberian ginseng* has demonstrated antitumor activity against murine melanoma [81]. The biosynthesized gold NPs fulfilled all the properties of the gold NPs, including spherical shape and crystalline nature. The SG-GNPs induced apoptosis of melanoma B16 cells through the generation of ROS and a decrease in mitochondrial membrane potential [82]. In another study, a combined treatment of chemotherapy (gold-paclitaxel NPs) and sonodynamic therapy (SDT) was employed against melanoma therapy. Initially, gold-paclitaxel NPs were synthesized and found to have 219.7±40.4 nm in size and negative zeta potential, indicating a higher degree of stability and good dispersion. These NPs showed promising cytotoxicity against C540 cancer cells in comparison to free drugs by generating more ROS and inducing apoptosis. When NPs were combined with SDT, a synergistic anticancer effect was observed, indicating that the gold NPs are excellent sonosensitizers [83].

Advanced melanoma patients or triple wild-type melanomas, having genotypes without mutations in the BRAF, NFI or NRAS genes, are completely neglected without effective and safe therapeutic options. Therefore, Gonçalves *et al.* [9] developed gum Arabic-functionalized Gold nanorods (GA-AuNRs), where gum Arabic improved biocompatibility and successfully stabilized the nanorods in the biological environment. These GA-AuNRs exceptionally reduced primary tumour growth by 45 % when tested in triple wild-type melanoma induced in mice. This result is attributed to the composite structure capacity of reducing melanoma cells' ability to invade the extracellular matrix and grow into colonies, thus, they can be safely and effectively used against subcutaneous tumours and metastasis.

Locally advanced melanoma has decreased 10-year survival rate from 24 to 68 % due to drug resistance and inefficient targeted chemotherapeutic agent delivery. To circumvent the above problem, Banstola *et al.* [84] fabricated anti-PD-L1 conjugated and doxorubicin-loaded gold nanoshells (T-HGNS-DOX). A significantly higher level of PD-L1 was expressed in the patient who died of locally advanced melanoma. These nanosystems showed exceptional in vitro and in vivo inhibition of the melanoma cancer model. When conjugated with an antibody and short chain of PEG on the surface of T-HGNS-DOX, it demonstrated improved precise targeting and localized doxorubicin delivery to melanoma cells. Thus, it can be concluded that following intratumoral administration of these nanoformulations led to their maximum retention and a considerable decrease in tumour growth due to elevated apoptosis markers and downregulation of angiogenesis and proliferative markers [85].

A novel and versatile theranostic nanoprobe comprised of gold nanocages labelled with 4-mercapto benzoic acid (p-MBA), followed by coating with liposomal layer and then conjugated with anti-MUC18 single chain antibody (scFy) was developed for melanoma diagnosis and selective treatment of melanoma cancer. The nanoprobe (p-AuNCs@scFv-lip) showed 24-fold cellular uptake by the A375 cell line than that of the SKBR3 cell line. This nanoprobe specifically identified A375 cells through surface-enhanced Raman spectroscopy and selectively killed A375 cells through the PTT principle. Thus, the nanoprobe would act as a great tool for in vivo tumour site tracing, biodistribution study and targeted treatment of cancer by combining chemotherapy and immunotherapy [86].

In one investigation, gold NPs coated with hyaluronic acid (HA) and oleic acid were developed and combined with PTT for the synergistic treatment of melanoma. When the biocompatible gold NPs with a mean size of 297 nm were used alone, they showed no cytotoxic effect on yeast and tested cell lines. However, gold NP administration followed by laser irradiation (750–1400 nm) showed a reduction of 20 % in melanoma cell line (B16F10) viability. Thus, this strategy seems to be effective against, in particular, against non-metastatic melanoma or other superficial tumours [87]. In another investigation, Shanei *et al.* [88] combined gold NPs conjugated folic acid (F-Cys-GNPs) with acoustic cavitation. The result showed decreased viability of melanoma cells at higher concentrations and sizes of NPs with adequate irradiation (frequency of 1 MHz). There was a significant decrease in side effects due to the presence of folic acid on the surface of NPs, which are specifically bound to folic acid receptors abundantly expressed in melanoma cells.

Nanocomposite comprised of gold NPs of 11.53±2.14 nm in diameter and TiO_2_ nanorods of 81.72±29.02 and 22.38±4.74 nm in length and diameter, respectively, was developed and combined with PTT and SDT separately and in combination for the treatment of melanoma. The Au-TiO_2_ nanocomposite was found to be highly stable and biocompatible and showed excellent photothermal conversion efficiency. Upon exposure to both PTT (lasers at 808 and 605nm) and ultrasound in the presence of 100 and 250 μg mL^-1^ Au-TiO_2_, intensive apoptosis and total ablation happened in melanoma cell line C540, resulting in the reduction of cell viability to less than 1 % [89]. Similarly, Soratijahromi *et al.* [90] developed a nanocomposite of gold NPs of 125±66 nm in diameter and manganese dioxide nanorods of 77±30 nm in diameter and up to 2 μm length as novel photosensitizer/sonosensitizer for PTT and SDT in the treatment of melanoma. Cytotoxicity of Au-MnO_2_ nanocomposite toward C540 cell lines was found to be increased in a dose-dependent manner. Intramural administration of a low dose of the nanocomposite in the melanoma-induced animal, followed by laser and ultrasound radiation, resulted in necrosis of the melanoma tissues.

##### Silver nanoparticles

Silver NPs are one of the most vital NMs among several NPs that play an important role in the treatment of cancer. This is because of their extensive reactivity, reduced size and spherical shape [91]. These properties enhance the bioavailability of therapeutic molecules after both systemic and local administration through penetration across biological membranes, distribution, and cellular uptake. Silver NPs acts by various mechanism against cancer tissues, including induction of apoptosis, metabolic changes, increasing oxidative stress, affecting membrane fluidity, regulating the cell cycle, autophagy in cancer cells and suppressing anti-apoptotic genes [92-94]. In addition, these NPs regenerate cell ATP, thereby causing increasing oxygen production and mitochondrial damage. Moreover, silver NPs stop the cell in the balancing phase of GM, which is attributed to DNA damage [95,96]. Silver NPs coated with anticancer agents can efficiently destroy cancer cells by delivering the drug to the targeted sites [97].

Based on the above fact, Zhao *et al.* [98] developed a spherical-shaped nanocomposite comprised of silver NPs and chitosan-starch biocomposite hydrogel. Silver NPs were monodispersed with particle sizes ranging from 5nm to 15nm and were uniformly distributed and stabilized by the hydrogel matrix. These NPs showed excellent antioxidant properties related to the quenching of free radicals. When these NPs were studied for anti-melanoma effect using six different types of malignant melanoma cell lines, the percentage of cell viability was found to decrease in a dose-dependent manner. Patil *et al.* [99] fabricated a multimodal theranostic agent with an organometallic system; silver NPs as metallic component provide photothermal ablation of cancer cells and pheophorbide-a(Ph-a) as an organic component provide chemotherapeutic and imaging activity. In vitro studies on cell lines demonstrated that these nanosystems caused apoptotic cell death due to PTT-mediated ROS formation. In vivo antimelanoma study showed effective suppression of tumour growth, which could be attributed to the combined effect of NPs and PTT.

In one investigation, a novel nanohybrid bio-hydrogel comprised of silver NPs embedded in carboxymethyl cellulose (CMC) and poly(acrylic acid-co-maleic acid (MA) was developed in the presence of octadecylamine (ODA) using a soft white LED approach (AgNPs@C-MA-O). The hybrid hydrogel was tested for its potency against human melanoma cells and the results demonstrated that the systems reduced viability of skin melanoma cells in a dose and time-dependent manner. The result was attributed to the generation of ROS and subsequent damage to cell or mitochondrial membranes, resulting in cell deaths. Further, the use of harmless white LED in the synthesis process enhanced the synergy between silver NPs and biopolymers and thereby enhanced the anticancer efficiency of the nanohybrid hydrogel [100]. Another nanohybrid hydrogel system with silver NPs embedded in cross-linking networks of CMC conjugated with doxorubicin was developed. The nanohybrid system was synthesized using a single-pot in-situ reduction of silver ions by CMC polymer (a green process), which is also used as a capping ligand and then electrostatic conjugation with doxorubicin. These systems showed tuned doxorubicin intracellular kinetic in vitro, indicating a synergistic effect with silver NPs in killing melanoma cells. In addition, this nanohybrid system also showed significant antibacterial activity against both gram+ve and gram-ve bacteria responsible for associated infection at the melanoma sites [101]. Valenzuela-Salas *et al.* [102] studied the antiproliferative and antitumor effects of silver NPs coated with polyvinylpyrrolidone (PVP) and compared the result with cisplatin activity. The coated silver NPs have an average particle size of 35±15 nm and metallic silver content of 1.2 wt%. Similar responses for antiproliferative potency and ROS production were observed for both coated silver NPs and cisplatin. However, significantly different cell death pathways were triggered, which was attributed to the induction of apoptosis and necrosis by cisplatin, while only apoptosis was induced by silver NPs. In addition, silver NPs showed a survival rate almost 4-fold higher compared with the survival rate observed with cisplatin. Moreover, the in vivo study on survivor mice treated with silver NPs did not demonstrate genotoxic damage with remarkable anti-melanoma activity.

A nanocomposite consisting of silver and platinum inorganic NPs was developed for its anticancer activity against melanoma. The anti-melanoma activity on the melanoma cell line was due to the effective interaction between these nanocomposites and cell membranes, killing the cancer cells and preventing metastasis. The selectivity of cytotoxicity of these NPs towards cancer cells could be attributed to the targeting of cancer cells through enhanced permeability and retention in these cells [2].

##### Copper nanoparticles

Compared to gold and silver, copper has a lower cost and can be developed through contemporary technologies, leading to enhanced development of cupper-based NPs as therapeutics. In addition, it was well documented that the cupper-based NPs demonstrated enhanced toxicity against microbes and fungi [103]. Copper NPs act against tumours by depleting intratumoral glutathione levels for enhanced ROS on account of the redox reaction between copper ions and glutathione [104,105]. In addition, copper peroxide (CuO_2_) composed of copper ions and peroxide groups, which have been employed for tumour-specific hydrogen peroxide (H_2_O_2_) self-supplying chemodynamic therapy (CDT) [106]. Other forms of inorganic copper-based NMs, such as CuS, CuCo_2_S_4_, and Cu_2_-xSe, have been used to create hyperthermia for the ablation of tumours [21].

Song *et al.* [107] investigated the potential of cuprous oxide NPs (Cu_2_O-NPs) against uveal melanoma cells. After application, these NPs adsorb serum protein in the cell culture medium, followed by their internalization by uveal melanoma cells *via* lipid-raft mediated endocytosis. These Cu_2_O-NPs not only inhibit cancer cell growth but also impair the migration and invasion ability of the uveal melanoma cell. The result was attributed to the damage caused by these NPs against mitochondria, lysosomes and autophagolysosomes, resulting in elevated ROS levels and, subsequently, apoptosis and autophagy of the uveal melanoma cells.

The tumour cell vaccine is a promising strategy to activate tumour immunity, which could be bone for cancer therapy. In this process, nanoenzymes can play an important role in catalysis-immunotherapy against cancer therapy. Artificial enzymes are preferred over natural ones as they can be prepared with more ease, are more stable in the preservation process, and can generate substances for tumour therapy *via* catalyzing chemical reactions in situ [108,109]. Considering the above fact, more recently, Fang *et al.* [110] developed a ferroptosis-activating vaccine employing artificial nanoenzyme copper telluride NPs (CTNPs) to induce catalytic immunotherapy. Firstly, mesoporous CTNPs were prepared, which have the ability to release copper ions to catalyze ROS accumulation and glutathione oxidation. This led to the triggering of ferroptosis and induction of immunogenic cell death for antigens and eliciting damage-associated molecular patterns. Thereafter, the CTNPs were loaded with ovalbumin (OVA) (CTNPs@OVA) as an exogenous antigen to boost the efficacy of endogenous antigens and induce a strong immune response in vivo. Further, CTNPs@OVA was coated with melanoma B16-OVA membrane (CM CTNPs@OVA) to avoid the separation of OVA from the surface of the nanosystem. Here, OVA directly induced the maturation of dendritic cells and subsequent T-cell recruitment. With the addition of PTT with the CM CTNPs@OVA administration, the function of ferroptosis-activating vaccines was enhanced along with the generation of strong immunotherapy against melanoma.

The common/traditional therapies such as surgery, chemotherapy and radiotherapy for melanoma suffer from various limitations, including serious systemic toxicity, large defects to the skin, high risk of recurring, undesirable treatment effectiveness and therapeutic resistance [14,15]. Therefore, alternative therapeutics such as PTT, PDT, immunotherapy, gene therapy, etc. are preferred. When administered intravenously, these therapeutic agents accumulate at the tumour sites in low concentration due to the poor targeting potential and intricate physiological barriers, resulting in subtherapeutic effects and severe side effects [111,112]. Thus, a robust and optimized administration approach must be adopted for tumour-targeted delivery. In this context, microneedle systems with minimally invasive, painless and efficient nature can fulfill the requirements. Based on this concept, Chen *et al.* [21] fabricated a microneedle system with PVP and CuO_2_NPs synthesized under an alkaline environment and stabilized by PVP. After the insertion of microneedles, CuO_2_NPs released and decomposed to Cu^2+^ and H_2_O_2_ in an acidic environment prevailed in the cancerous tissues. Thereafter, Cu^2+^ could transform endogenous H_2_O_2_ into highly toxic OH for CDT, further enhanced by self-supplied H_2_O_2_. In addition, CuO_2_NPs could utilized as a glutathione-scavenging agent for boosting ROS-induced tumour cell death. This was confirmed *via* an in-vitro study where CuO_2_NPs encapsulated microneedles were able to kill melanoma cells by self-enhanced CDT. Being a photosensitizer, CuO_2_NPs demonstrated hyperthermia in the melanoma area in vivo under the irradiation of NIR. Furthermore, the microneedle system showed minimal side effects in vivo under combination therapy.

##### Platinum nanoparticles

Most recently, platinum NPs have attracted the attention of many researchers because of their unique physico-chemical properties such as ease of synthesis, small tuneable size, high surface area for functionalization for high drug loading, and platinum NPs and platinum-based compounds demonstrate dual functionality [113-115]. In addition, platinum-containing anticancer drugs such as carboplatin, cis-platin and oxaliplatin have been used widely for the treatment of cancer [116,117]. Further, it has a scavenging effect against superoxide and H_2_O_2_ due to its anti-oxidant properties. The mechanisms involved in the anticancer property of platinum NPs include the activation against signalling pathways p21 and p53, resulting in apoptosis and proliferating cell nuclear antigen-mediated growth arrest. As per a recent report, silver-platinum NPs have huge potential against melanoma and glioblastoma cells, indicating that the NPs showed cell membrane interaction, killing cancer cells in addition to cancer metastasis [2,118].

Mukherjee *et al.* [113] designed and developed PEG-assisted/stabilized colloidal Pt-NPs employing the borohydride reduction method. This nanosystem showed exceptional stability at room temperature (2 years) and serum and pH 7.4 phosphate buffer (one week) and biocompatibility in vitro (normal cell line) and ex vivo (chicken embryonic model). The resulting nanosystem was converted into a drug delivery system (DDS) by conjugating with doxorubicin. This DDS exhibited inhibition of cancer cell (B16F10 and A549) proliferation and induced apoptosis of cancer cells. Intraperitoneal administration of the DDS gave a considerable reduction of tumour growth in the subcutaneous murine melanoma tumour model compared to a control group with a free drug. The mechanism was attributed to the possible involvement of the p53-mediated apoptotic signalling pathway and downregulation of SOX2 and Ki-67 proliferation markers.

Salehi *et al.* [119] developed platinum mesoporous NPs with particle size and pore size <11 nm and 5 nm, respectively, and evaluated their toxicity against melanoma cell line C540 alone or combined with laser radiation and X-ray irradiation. When used alone, these NPs showed low toxicity against cell lines as well as laser radiation (no toxicity) and X-ray irradiation (limited toxicity). However, the combined therapy (laser radiation followed by X-ray irradiation) demonstrated deep cell killing with a very low melanoma cell viability (r1 %), indicating a synergistic activity. In addition, these NPs acted as potential photosensitizers and radiosensitizers. The mechanism was attributed to the significant generation of ROS upon combined exposure of the cell line to laser and X-ray.

##### Silica nanoparticles

Silica or silicon NPs were invented in the 1960s and widely used as catalysts because of their large surface-to-volume ratios. Silica NPs are one of the most popular and common inorganic NPs to be used for special biological applications due to their suitable physical parameters such as size, shape, tuneable pore size (2 to 10 nm) for drug encapsulation [73], ordered pore structure, ease of camouflage by chemical conjugation with biocompatible, targeting and imaging ligands [120]. The applicability of silica NPs increased manifold due to the synthesis of mesoporous silica NPs in the 1990s [72], which have superior quality than other inorganic NMs in terms of their biocompatibility, degradability and drug release rates. Mesoporous silica NPs provide an effective nanocarrier DDS for the oral route due to their hydrophilic surface leading to higher affinity for the head groups of different phospholipids and can be modulated employing various surfactant concentrations, templates pH and solvents during their synthesis, high drug loading capacity, high cellular penetrations, protection from the acidic environment of the stomach and most important of all is the controlled release with targeted delivery [5]. It was reported that mesoporous silica NPs have been used as gatekeepers for controlled drug release [121,122], and found to be endocytosed by tumour cells, macrophages as well and non-cancer cells [123].

The importance of plant-derived compounds has been recognized as potential agents for the prevention and treatment of different types of cancers, including melanoma. Among many plant compounds, curcumin has been proven to be a promising anticancer compound [124]. Therefore, Ghazaeian *et al.* [125] fabricated curcumin-silica nanocomplex as a photosensitizer for PDT in the treatment of melanoma. The curcumin loading onto silica was found to be successful, leading to the enhancement of curcumin solubility in water. The nanocomplex showed interaction with DNA but no interaction with haemoglobin. The investigation of curcumin nanocomplex and PDT on human melanoma cancer cells (A375) and human fibroblast cells demonstrated higher cancerous cell death compared to free curcumin. The mechanism was attributed to the formation of ROS, which contributes to cell death [125]. Further, Rizzi *et al.* [28] employed the same mesoporous silica NPs (M SiNPs) to conjugate with second-generation photosensitizer verteporfin to form nanocomplex (Ver-M SiNPs). The formed nanocomplex was tested in PDT therapy against melanoma and the results showed that the nanocomplex selectively reduced cancer cell proliferation in highly invasive melanoma cell line (SKMEL-28) without affecting the proliferation of either a normal human keratinocyte cell line (HaCaT) or low metastatic melanoma cell line (A375P), thus assuring lower side effects. The main issue remaining with conventional PDT is the limited penetration depth of visible light into the tumour tissue essential for its activation. To improve upon it, mesoporous-silica-coated upconversion fluorescent NPs (UCNs) were developed by Idrish *et al.* [126], which acted as carriers for photosensitizer and as a transducer in the conversion of deeply penetrating NIR light to visible wavelengths. A greater PDT efficacy with the dual photosensitizer strategy in comparison to single photosensitizer due to the improved formation of ROS and reduced cell viability. An in vivo study on tumour-bearing mice demonstrated that there was tumour growth inhibition in PDT-treated mice by intravenous injection of UCNs conjugated with a tumour-targeting agent or direct injection of UCNs into melanoma tumours.

In an attempt to enhance the delivery of therapeutics and give precise targets to disease sites, Kim *et al.* [127] employed Cornell dots, core-shell silica NPs, where SiNPs were coated with PEG (PEGylated SiNPs) and functionalized with conjugating melanoma-targeting peptide α_v_β_3_-integrin to PEGylated SiNPs. The ultrasmall (<10 nm in diameter) PEGylated SiNPs with targeted peptides induced ferroptosis in starved cancer cells and tumour xenografts in mice when injected intravenously. A nano-platform for both PDT and chemotherapy was fabricated with a gold nanoshell on rod-like mesoporous silica NPs with different aspect ratios. Cellular uptake and distribution of NPs in tumour tissue were found to be significantly enhanced with aspect ratio, and at moderate aspect ratio, the gold nanoshell was able to penetrate melanoma tissues, resulting in the potential ablation of malignant melanoma cells in a single treatment [128].

To effectively trigger pyroptosis at the melanoma site and to enhance the therapeutic efficiency, a distinct 2D core/shell nanosystem comprised of engineered mesoporous silica layer and silicene with loaded cisplatin was designed and fabricated to achieve synergistic pyroptosis and hyperthermia of melanoma carcinoma. The surface-nanopore engineering strategy was adopted to induce surface functionalization of a 2D silicene layer that enhanced hydrophilicity, provided abundant chemical groups for target modification and, more importantly, on-demand drug delivery. Upon NIR-irradiation, the 2D silicene core generated hyperthermia, resulting in cisplatin release for activating caspase-3, cleaving gasdermin E (a protein-coding gene, CSDME) into the GADME-N terminal and further inducing pyroptosis. Both in vitro and in vivo studies showed that the nanosystem has active targeting potential into tumours and synergistic pyroptosis (release of cisplatin and hyperthermia), thus significantly eradicating melanoma without any adverse effect [129]. Sapino *et al.* [130] functionnalized the mobil composition of matter no 41 (MCM-41), which is a mesoporous material with a hierarchical structure composed of silicate and alumosilicate, with the aminopropyl group and then loaded it with flavonoid quercetin. The NP system was found to improve the stability of quercetin and enhance quercetin penetration across the skin. Further, the nanosystem showed inhibition of cancer cell proliferation in melanoma cell line JR8.

##### Titanium dioxide nanoparticles

TiO_2_ has been widely used in dermal creams, lotions, sun protection creams and other cosmetics for the protection of skin against UV light that has the potential to cause melanoma. This is feasible due to its biocompatibility, penetration ability when the size is 5-20 nm, reactivity and chemical stability. TiO_2_ NPs can be used as anticancer agents because of their high accumulation in cells, resulting in modification in gene expression, inflammatory responses, oxidative DNA damage, and lipid peroxidation, leading to necrosis or programmed cell death [69,131]. In addition, TiO_2_ NPs are most often used in phototherapy as they exhibit excellent optical, electrical, magnetic, photocatalytic, structural stability and biocompatibility [132,133]. Being a photoactive agent, TiO_2_ NPs act as anticancer agents through activation through light and ultrasound waves, promoting the generation of heat or ROS [134].

Curcumin is a well-established therapeutic agent, but its clinical application is limited because of its hydrophobic nature, resulting in low bioavailability. Thus, TiO_2_ NPs were initially coated with chitosan to enhance their stability and biocompatibility and then curcumin molecules were conjugated onto them to form nanocomposite (TiCurNC). These nanocomposites showed exceptional selectivity towards the melanoma cell line (B16F10) while minimizing toxicity to normal cells (CHO–K1 cells). The potent anticancer activity against melanoma cells was due to the increased intracellular content of curcumin, followed by the induction of mitochondria to produce more ROS, which interacts with the DNA and causes its damage and apoptosis. In addition, it is responsible for stopping the cells at the G2/M phase [135].

##### Iron oxide nanoparticles

Iron oxide NPs or magnetic NPs referred to as Fe_3_O_4_ and Fe_2_O_3_ were first explored in drug delivery by Widder *et al.* [136] in 1979. These NPs are approved by the FDA for therapeutic and imaging use due to their controlled size, possibilities of surface modification, extremely low toxicity, magnetic responsiveness and contrast agent for magnetic resonance imaging [137]. When induced by external electromagnetic fields, iron oxide NPs play a crucial role in their transfection and delivery along with the conjugated or coated therapeutic agents [138]. Magnetic medication delivery minimally comprises an inorganic material core (iron oxide NPs) and a surface coating in order to enhance biocompatibility, half-life and stability [139]. For instance, polymer (e.g., dextran, chitosan) or organic surfactant (e.g., sodium oleate) coating of iron oxide acts as a barrier and thus prevents reticuloendothelial system absorption and NP agglomeration [140]. Reduction of particle size of iron oxide below 100nm may lead to thermal energy equal to its anisotropy energy and exhibit superparamagnetic nature. This property of iron oxide NPs enables them to heat up under the influence of magnetic fields to induce hyperthermia and subsequent thermal ablation of tumours [72].

Polyphenols are plant-derived constituents that can self-assemble with metal ions to form a metal-polyphenol network (MPN) through a chelate reaction [141,142]. The qualities such as rapid and green synthesis, low cost and pH responsiveness make MPN a potential candidate for coating biomedicals [143]. Based on the above concept, Mu *et al.* [144] employed MPN to coat cabazitaxel (Cab@MPN) *via* the coordinate reaction between metal and polyphenol. The drug loading efficiency was found to be increased from 7.56 % to 9.28 % after the introduction of chitosan coating (Cab@MPN/CS). The protonated amines and swelling process of chitosan led to the faster release of cabazitaxel in the acidic environment of melanoma. The chitosan coating provided positive charges to the surface of NPs, which improved internalization in B16F10 cells and then Cab@MPN/CS escaped lysosomes *via* proton sponge effect. Now, the released cabazitaxel acted on microtubules and induced cell apoptosis. In vivo study showed that the Cab@MPN/CS NPs have longer retention time in tumour tissues and significantly reduced tumour growth by upregulating TUNEL expression and downregulating K167 and CD31 expression. On a similar concept, a nano-delivery system (nanocomposite) comprised of iron (Fe^+3^) based MPN with gallic acid grafted HA (Ce6@HA-GA NPs) was developed. Here, Chlorin e6 (Ce6), a second-generation photosensitizer, was used. The intracellular ROS generation for Ce6@HA-GA NPs *via* CDT and PDT was higher than Ce6-induced PDT and Ce6@HSF NPs-induced CDT. Both in vitro and in vivo studies revealed that Ce6@HA-GA NPs had the best anti-melanoma effect [145].

Most recently, Li *et al.* [146] successfully developed an injectable nanocomposite alginate-graft-dopamine (SD) hydrogel with entrapped biomimetic polydopamine-Fe(III)-doxorubicin NPs (PFD NPs). The nanocomposite hydrogel was capable of delivering doxorubicin precisely to the tumour site and thereafter PDT-based killing of cancer cells mediated by PFD that converted NIR irradiation into heat energy. In addition, PFD can catalyze excessive endogenous H_2_O_2_ into O_2_ to relieve tumour hypoxia. Thus, this nanocomposite provided synergism through PDT, chemotherapy and nanozyme therapy. Both in vitro and in vivo studies revealed that nanocomposite hydrogel can significantly inhibit the proliferation and migration of melanoma cells. In addition, this hydrogel can significantly enhance epidermal regeneration through bactericidal and ROS scavenging effects and increase the proliferation and migration of cells.

#### Carbon-based NMs

Sumio Iijima of Japan first invented carbon nanotubes (CNTs) in 1991 [147]. These comprise big cylindrical molecules with a hexagonal arrangement of sp2 hybridized carbon atoms. The CNTs showed exceptional tensile strength, higher conductivity, and excellent thermal and chemical stabilities [148-150]. CNTs are made up of the rolling of a single-walled graphene sheet called single-walled CNTs (SWCNTs), whereas the rolling up of more than one graphene sheet constitutes multi-walled CNTs (MWCNTs). SWCNTs are preferred as drug delivery vehicles due to their distinct wall and small width. However, MWCNTs are characterized by defects in their structure, resulting in poor stability, but on the other hand, they provide opportunities for modification [151]. Drug molecules can be loaded onto CNTs through the following interaction: entrapments inside CNT channels or within CNT bundle or CNT mesh and attachment of the drug molecules to the outer wall of CNT through functional groups [152]. Thus, CNTs are widely researched as an effective delivery vehicle for a variety of drugs and photosensitizing agents [153].

A versatile SWCNTs-based nanovector was developed for the delivery of doxorubicin to treat melanoma. Here, the SWCNTs were modified to conjugate a doxorubicin prodrug (SWCNT-DOX) *via* a carbamate linker, which cleaves enzymatically to cause temporal release of the active drug. The resulting nanovector induced time-dependent death of B16-F10 melanoma cells *in vitro*. The SWCNT-DOX NPs internalized immediately into the lysosome of melanoma cells and were retained subcellularly for over 24 h. *In vivo* melanoma model treated with SWCNT-DOX nanovector ceased the tumour growth without producing systemic side effects associated with doxorubicin [154].

Low-intensity ultrasound irradiation with sonosensitizer has shown exceptional advantages for cancer therapy due to its advantages, including penetration to deeper tumours, invasiveness, and targeted uptake without serious side effects compared to other cancer therapies. Based on this, a nanocomposite comprised of polypyrrole (sonosensitizer)-coated MWCNTs (PP-MWCNTs) was fabricated and employed in melanoma therapy. In vitro investigation on the nanocomposite showed a concentration-dependent cytotoxicity with a cell viability of 8.9 % in multi-step ultrasound irradiation. This four-step SDT therapy resulted in efficient temperature increment in both the C540 (B16/F10) cell line and a melanoma tumour model in male balb/c mice. Histological analysis and tumour volume decrease after 10 days showed 75 % necrosis and 50 % decrement, respectively [155].

##### Graphene-based NMs

Graphene-based NMs were isolated first from a bulk graphite. These NMs are classified into various types, including graphene with varied layers, graphene oxide (GO) and reduced GO (rGO) [156]. However, GO and rGO have been potentially used in biomedical and pharmaceutical fields due to their easy availability, high biocompatibility, and photothermal and tuneable physicochemical properties [157]. As far as cancer therapy is concerned, GO and rGO are being used either as drug delivery vehicles for anticancer drugs or as photothermal agents [158].

##### Graphene oxide

Graphene oxide (GO) is synthesized from graphite through various methods, such as the Hummers method [159], the Brodie method [160], and the Staudenmaier method [161], using various chemical reagents [157]. The basic principle in converting graphite to GO involves exfoliation and oxidation. The puckered sheets of GO are exfoliated and then oxidation of a basal plane resulted in the introduction of different functional groups such as hydroxyl, epoxy and carboxyl [162]. These oxide groups confer GO the hydrophilicity and the potential to improve the solubility of poorly water-soluble drugs and improve drug absorption and mechanical strength [67,163]. In addition, GO can be functionalized through covalent bonds with diverse classes of drugs under mild experimental conditions.

Initially, rGO was synthesized as an alternative material to pristine graphite as it can be produced in large amounts [164]. Basically, rGO is synthesized from GO through various methods such as thermal, chemical and electrochemical reduction [165]. Based on the synthesis method, the properties of rGO varied in terms of structure, thermal, electrical and mechanical properties [166]. The oxygen-containing groups cannot be removed completely and residual oxygen groups provide rGO the opportunities to be modified with different functional or conjugated with different drugs. In addition, there is a reduction in graphene layer distance and thickness because of the reduction in branched groups [157].

A novel nanocomposite hydrogel DDS was developed to treat melanoma and associated pathogenic infections, which generally aggravate the melanoma tissue. Herein, the anticancer drug fluorouracil (5FU) was loaded onto rGO and then this nanodrug was loaded into a polymeric matrix of functionalized arabinoxylan (carboxymethylarabinoxylan, CMARX) extracted from *Plantago ovata*. The CMARX loaded with rGO-5FU was then cross-linked with tetraethylorthosilicate to form nanocomposite hydrogel (hydrogel rGO-5FU-CMARX system). These hydrogels showed pH sensitivity with different swelling and biodegradation under different pH conditions, resulting in sustained release of loaded 5FU. In vitro study revealed that this nanocomposite hydrogel has significant anticancer and antibacterial activities against the U87 cell line and *S*. *aureus* and *P. aeruginosa*, respectively [167].

A novel nanocomposite sheet was developed from chitosan oligosaccharide-grafted nano-GO (nGO-COS), modified with CD47 antibody and loaded with dacarbazine (DCB) (nGO-COS-CD47/DCB) for synergistic targeted chemo-photothermal therapy against melanoma. COS enhanced the biocompatibility of the nanocarriers and also provided an active interface for modification with CD4 antibody. The anticancer drug dacarbazine was loaded onto the nanocomposite *via* high capacity by π–π stacking. The prepared nanocomposite drug carrier showed exceptional biocompatibility, photothermal conversion efficiency, precise targeting, and fast drug release under NIR irradiation, followed by efficient apoptosis of cancer cells. The CD47 antibody enhanced the targeting capability of the nanocomposite against melanoma cells. The nanocomposite was able to release the drug efficiently, induced by both exogenous (PDT) and endogenous (acidic environment in cancerous tissue) activation to cause the death of B16-F10 melanoma cells. The nGO-COS-CD47/DCB nanocarriers (chemotherapy) and NIR irradiation (PDT) simultaneously induce apoptosis through the mitochondria apoptosis pathway in vitro [168].

More recently, Bamburowicz-Klimkowska *et al.* [169] synthesized next-generation NMs graphene encapsulated magnetic NPs (GEMNs) for the delivery of genes into melanoma cells. In this study, a green fluorescence protein expression plasmid DNA (pDNA) was employed and GEMNs were decorated with branched polyethyleneimine (PEI) to synthesize nanotransporter (GEMNS-PEI/pDNA. The nanotransporter was capable of condensing p-DNA, leading to their efficient delivery into the melanoma cells (B16F10).

##### Nanodiamonds

Nanodiamonds are carbon-based NMs with biocompatibility and easily modified surfaces. Thus, nanodiamonds are considered a promising candidate in therapeutics. Gou *et al.* [170] synthesized carboxylated nanodiamonds and studied tumour cell migration inhibition. The carboxylated nanomedicine enhanced single-cell adhesion and impaired assembly of the skeleton. These NMs showed remarkable inhibition of the migration of Hela cells by preventing the epithelial-to-mesenchymal transition process *via* the transforming growth factor β signalling pathway (TGF-β). In addition, these NMs demonstrated potential reduction of the metastasis of murine B16 melanoma cells in an in vivo pulmonary metastasis model.

### Organic NMs

**Figure 4.**
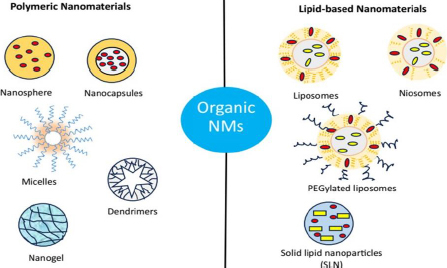
Illustration of different types of organic NMs: polymeric NMs and Lipid-based NMs

Organic NMs are the most thoroughly studied and applied as one of the most attractive drug delivery vehicles due to their innate and significant characteristics such as biocompatibility, biodegradability and ease path of drug molecule encapsulation [171]. Organic NMs are generally comprised of polymers and lipids, which can be designed into different suitable structures to increase payload capacity and controlled release of the payload at the targeted site, leading to the significant enhancement of the therapeutic efficiency of the loaded drug with minimized side effects. In addition, many organic NMs exhibit stimuli-responsive properties that give impetus to the site-specific drug delivery more precisely to the cancerous tissues [172,173]. By linking desired functional groups, organic NMs can be employed for active or targeted drug delivery capabilities. Organic NMs include polymeric, micelles, dendrimers, nanogel, nanocrystals, nanoemulsion, liposomes, niosomes, SLN and exosomes (Figure 4).

#### Polymeric NMs

Polymeric NPs are considered a versatile category of nanocarriers that are widely employed in controlled and site-specific drug delivery [174]. These are classified into three generations: (i) the first generation of polymeric NPs has been used to encapsulate the active ingredients and provide their sustained release, (ii) the second generation of polymeric NPs focussed on the strategies to deliver the drugs in the target sites by incorporating stimuli-responsive properties, and (iii) polymeric NPs of third generation represented by multi-functionalities including targeting along with multi-drug release properties [175]. Various physicochemical properties of polymers, including size, shape, surface charges, flexibility, and length of the carbon chain, have been readily explored for the enhancement of characteristics of polymers such as high drug loading, biodegradability, biocompatibility, reduction in immunological reaction, ease of surface modification [176], functionalization with ligands for the improvement of binding affinity to specific receptor or cancerous cells [177].

Based on the source of origin, polymers are of two types: synthetic polymers (e.g., poly(lactic acid) (PLA), poly (glycolic acid) (PGA), poly(lactic-co-glycolic acid) (PLGA), poly(g-caprolactone) (PCL) etc.) and natural polymers, including alginate, chitosan, gelatin, collagen, dextran, heparin and albumin [178]. Depending on the methods of preparation, polymeric NPs are either nanocapsules or nanospheres. Nanocapsules have core-shell structures with the drug encapsulated inside a polymeric shell coat or membrane, while nanospheres are matrix-type structures where the drug is dispersed throughout the polymeric matrix [179]. They can also be classified into two general categories, such as homopolymers (formed with the same monomers) and copolymers (developed from different monomers). In this part, we will be dividing polymeric NPs into two categories based on their constituents, including nanospheres, nanocapsules, micelles, dendrimers, nanogel, nanocrystals and lipid-based NPs such as nanoemulsions, liposomes, niosomes, SLN and exosomes

#### Polymeric nanospheres

Nanospheres are spherical and uniform-sized particles and comprise either biodegradable or non-biodegradable polymers. The drug molecules are evenly distributed throughout the polymer matrix or adsorbed on the surface, resulting in a regulated release pattern [180]. The general method of preparation involves the solubilization of monomers and drugs in a suitable solvent, followed by the induction of cross-linking of monomers, thus entrapping the drug molecules inside the matrix [25]. These NPs allow surface modification to improve their drug-targeting efficiency.

#### Polymeric nanocapsules

Polymeric nanocapsules are core-shell structures with the active drug moiety inside the core or reservoir in solid, oily or molecular dispersion. This core is surrounded by a polymeric coating or a membrane [181]. The cavity or the core allows a high payload of the drugs and the coating provides a predetermined rate of drug release. They are developed employing methods such as interfacial deposition, layer-by-layer method, nanoemulsion, etc. Nanocapsules are widely used in the treatment of skin cancer therapy due to their merits, such as lower irritation scores and enhanced stability [182].

In order to improve upon the broad applicability, poor water-soluble curcumin was conjugated with hydrophilic copolymer composed of phenylboronic acid (PBA) and 2-aminoethylmethacrylate (AEMA) *via* covalent linkage between the 1,3-diketone group in curcumin and PBA group. This stable conjugated NPs (approximately 400 nm) at pH around 7.4 and underwent immediate decomposition when the pH reached around 5.5 to release the conjugated curcumin. These NP systems selectively killed more than 90 % of human malignant melanoma cells (A375), while around 70 % of the mice-derived fibroblast cells (L929) survived [183]. Hybrid NPs with polymer core (PLGA) and lipid shell composed of hydrogenated soy phosphatidylcholine, cholesterol and 1,2-disteroyl-sn-glycero-3-phosphaethanolamine-N[succinyl (PEG)-2000, followed by vitamin D3 functionalization was synthesized to target vitamin D receptor expressed on melanoma cells. The hybrid NPs showed initial burst release within 24 hours and thereafter diffusive transport in vitro. They were found to target B16 melanoma cells efficiently and thus can act as a potential carrier to deliver therapeutics to treat melanoma [184].

More recently, Song *et al.* [185] fabricated polymeric NPs with IL4RPep-1-poly-(lactic-co-glycolic acid)-grafted poly(ethylene glycol) (IPP) copolymer, loaded with chemotherapeutic agent celecoxib and immunotherapeutic agent afuresertib to improve bioavailability and to minimize side effects when administered without nanocarriers. The NP system is comprised of three parts: a hydrophobic core of PLGA for drug loading, a hydrophilic corona of PEG and a targeting peptide IL4RPep-1 which improves the targeting potential by binding to IL-4 receptor antigen on the surface of melanoma tumour cells. These IPP NPs were accumulated in the melanoma cells *via* EPR and showed an afuresertib-mediated reduction in expression and phosphorylation of protein-kinase-B in tumour cells. This led to the enhancement of apoptosis of melanoma cells and the precise regulation of M2 macrophages in tumour cells to reprogram M1 macrophages resulting in the enhancement of immune response and inhibition of tumour growth by celecoxib. Thus, the polymeric NPs delivered chemoimmunotherapeutic agents employing both spatial and temporal networks and can be great assets for the treatment of ectopic melanoma.

#### Dendrimers

Dendrimers are 3D spherical, highly branched, unimolecular, micellar structures having a size of around 20 nm [186]. Basically, the dendrimer structure comprises three distinct regions: a core or a focal moiety, a well-defined regularly branched symmetrical repeated structure originating from the core and functional end-groups on the outer layer of repeated units [187]. These NPs have numerous advantages over other nanocarriers, including vast internal cavities and a higher degree of branching, allowing high drug payload, structural feasibility in size and molecular weight, and monodisperse properties along multivalent characteristics [50]. The flexibility of adjustment of critical properties such as size, surface topology, architecture, solubility and chemical constitution make dendrimers a versatile nanocarrier. In addition, they are capable of entrapping both hydrophilic and lipophilic drugs due to the presence of a hydrophilic surface and hydrophobic core. Moreover, drugs can be physically encapsulated into the void space or covalently bonded on the surface with functional groups [188]. Biocompatibility and cancer specificity can be incorporated into dendrimers through surface modification and attaching suitable ligands. They are capable of delivering the loaded drugs in a controlled manner and at the specific site. Dendrimers are internalized through cellular endocytosis, thus promoting the transit of medicines through different types of cell membranes or biological barriers [5].

Jiang *et al.* [189] have fabricated polyamide amine dendrimer (PAMAM)-PEG-GE11-HA conjugates loaded with temozolomide for their targeting potential towards melanoma cells. Here, GE11 acted as an active targeting agent in antitumor therapy. This dendrimer-based nano-DDS was developed by ultrasonic emulsification and demonstrated suitable characteristics such as a mean particle size of 183.2 nm, a regular sphere with good uniformity, zeta potential of -0.01 mV, entrapment efficiency of m50.63 % and drug loading of %10.4 %. This nano-DDS showed potential targeting towards A375 human melanoma cells with minimal absorption into the normal cells.

Most recently, Russi *et al.* [7] investigated the targeting of BRAF in melanoma patients with BRAF inhibitors dabrafenib and vemurafenib by successfully encapsulating them into core-shell nano-micelles of an amphiphilic dendrimer-based on G2 PAMAM head and C18 aliphatic chains. Encapsulation of BRAF inhibitors in dendrimer was found to improve tissue permeability and extend the systemic drug circulation, extended-release at the targeted site, thereby reducing systemic toxicity associated with chemotherapeutics. Developed nano-micelles (m10 nm) showed excellent encapsulation efficiency with good drug-loading ability. Further, the release of both drugs from nano-micelles was enhanced at an acidic pH of 5.0 in vitro. When tested on melanoma cell lines, both drugs showed enhanced response in comparison to free drug treatment.

#### Polymeric micelles

Polymeric micelles are nanoscale (10-100 nm) core-shell structures comprised of spontaneous self-assembly of individual di/tri-block copolymers in aqueous medium [186]. The structure has a lipophilic core that can entrap hydrophobic drugs and a hydrophilic shell, generally formed of PEG, which prevents the aggregation of micelles and helps prolong the circulation time in the blood [190]. Polymeric micelles possess characteristic features such as nano-size, appropriate surface chemistry for attachment of ligands and functionalization, sufficient accumulation at the tumour site due to EPR effect and provide safe, concurrent or sequential consecutive IV infusion in diverse treatment including an aggressive form of tumour [3]. Despite all positives, these micelles show burst drug release and lack of targeting ability. A stimuli-responsive polymeric micelle structure was developed to circumvent the issue of burst release of active drugs [72]. On the other hand, to enhance the target specificity and binding ability, the corona (surface of the micelle) can be conjugated with mAb, folate monosaccharides, etc. [191].

A novel smart and efficient polymeric micelles-based nano-DDS embracing a combinatorial approach of chemotherapy and PDT was developed to target multiple independent therapeutic pathways in the treatment of melanoma. The polymeric micelles were prepared from methoxy poly(ethylene oxide) and PLGA block copolymer (mPEG-b-PLGA) loaded with DOX and IR-768 photosensitizer with a hydrodynamic diameter of around 25 nm and spherical size. These micelles demonstrated targeted delivery and accumulation in mitochondria of A375 melanoma cells through the EPR effect and contributed to excellent singlet oxygen generation capacity for PDT. The combination approach reduces drug dose and minimizes side effects towards normal HaCaT keratinocytes and triggers synergistic anticancer effects in melanoma cells [192].

#### Nanogels

Hydrogels are a three-dimensional cross-linked network of polymers with high water imbibing capacity and swelling. When hydrogels are formed in the nanoscale range (100-200nm), they are called nanogels [193]. Nanogels showed several potential advantages over other nanocarriers, including control over size, large surface area, enhanced stability due to enclosed drugs, higher drug loading, controlled release of active ingredients, and enhanced stimuli-responsiveness to external stimuli such as temperature, pH, ions, and enzymes [194,195]. Nanogels can be developed *via* various techniques such as chemical cross-linking, polymerization of monomer units, physical self-assembly and template-associated nanofabrication [196]. Based on the requirements, there may be simple, core-shell, multi-layer, hollow or functionalized types of nanogels.

Glucose oxidase (GOX) acts upon glucose to convert it to gluconic acid and H_2_O_2_, which has synergistic potential for cancer-starving and oxidation therapy. Based on the concept, Zhao *et al.* [197] developed a glucose-responsive nanomedicine, GOX-polymer nanogels, comprised of GOX and polymer 4-formyl-N-(3-(2-(2-(3-methacrylamidopropoxy)ethoxy)ethoxy)propyl) benzamide (FBMA) and oligo-ethylene glycolmonomethyl ether methacrylate (OEGMA) (poly(FBMA-co-OEGMA). This nanogel showed glucose-responsive H_2_O_2_ generating potential in vitro, improved stability and significantly improved tumour retention than that of GOX alone. In addition, the nanogel showed high anti-melanoma efficacy without systemic toxicity due to the confinement of GOX to only the tumour region.

An injectable thermosensitive nano-hydrogel was developed with cationized agarose and TMPO-oxidized lignocellulose at different proportions and loaded with PP-NPs containing deferasirox, an inhibitor of iron metabolism in the cancer cells by chelating action. The nano-hydrogel containing 2 % lignocellulose and 0.5 % agarose demonstrated the shortest gelation time (10.3 min) with the highest mechanical strength. This nanogel demonstrated dose-dependent cytotoxicity and this efficacy was further increased by irradiation with laser light [198].

#### Lipid-based NMs

Bangham *et al.* [72] first introduced lipid-based NMs for drug delivery in 1965, initially called “bangosomes” and then liposomes. Lipid-based NPs are the most popular drug organic nanocarriers for cancer treatment due to their biodegradability, biocompatibility, simple synthesis techniques and higher drug-loading efficiency [199,200].

#### Liposomes

Liposomes are spherical vesicles comprised of an aqueous core enclosed by one or more lipid bilayers. This enables the loading of hydrophilic drugs at the aqueous core and lipophilic drugs in the lipid bilayers [201]. They are mostly synthesized from one or more phospholipids such as phosphatidylcholine (PC), phosphatidylethanolamine (PE), phosphatidylglycerol, *etc.*, and cholesterol as a stabilizer for the bilipid layer [202]. Liposomes as DDS have many advantages, such as nanoscale size, amphiphilic nature, biocompatibility, ease of preparation through numerous methods, and exceptional endocytosis and diffusion, leading to their interactions and penetration across biological membranes [203,204]. However, the higher production cost, short half-life, and stability issues like leakage leading to premature drug release and opsonization after administration through the IV route are the major issues with liposomes [205]. Pegylated liposomes (PEG molecule coated liposomes) can circumvent the problem of opsonization, resulting in long circulation time of liposomes. Similarly, the incorporation of cholesterol prevents the leakage problem by increasing the phase transition temperature of phospholipids [206]. Other types of liposomes developed are cationic liposomes, pH-sensitive and immune liposomes, which have great importance in targeted drug delivery.

To improve upon the limitation of cancer vaccine efficacy, a combinatorial approach with liposomal celecoxib and ex vivo generated dendritic cell vaccines pulsed with gp100 peptide was undertaken for the prophylactic and therapeutic anti-tumour immune response. The IV administration of liposomal celecoxib in a mouse model of melanoma showed the normalization of the tumour microenvironment by inhibiting the proliferation of regulatory T cells and IL-10 production. The combination of liposomal celecoxib and dendritic vaccine pulsed with gp100 peptide significantly improved CD4+ and CD8+ T cells infiltration and secretion of IFN-γ. Thus, the combinatorial strategy successfully demonstrated tumour growth inhibition and overall survival [207].

In one study, chitosan-coated liposomes were developed with the intention to stabilize and increase the skin permeation potential of Indocyanine green for PDT against melanoma. No doubt, the prepared liposomes protected indocyanine green from degradation as compared to uncoated liposomes. The skin permeation of indocyanine green was found to be significantly increased by chitosan-coated liposomes. In addition, the cellular uptake and photocytotoxicity of indocyanine green in the melanoma cell line (B16-F10) were exceptionally high for the coated liposomes in a chitosan-dependent manner [208]. In another study, dacarbazine and eugenol-loaded liposomes were surface-functionalized with HA for the treatment of aggressive metastatic melanoma. The obtained coated liposomes showed higher cytotoxicity of 95.08 % at a dacarbazine concentration of 0.5 μg mL^-1^ compared to only 10.20% cytotoxicity shown by dacarbazine solution at the same concentration level. In addition, the number of late apoptotic cells was found to be much higher for the coated liposomes at 45.16 % than that of the dacarbazine solution (8.43 %). Moreover, the coated liposomes demonstrated significantly higher inhibition of cell migration and proliferation, indicating their ability against metastasis. Eugenol was found to downregulate surviving protein, thereby allowing dacarbazine to function in an efficient manner [209]. In a separate study, 5FU-loaded liposomes were developed and subsequently surface functionalized with AS1411 aptamer for the treatment of basal cell carcinomas. The functionalization of liposomes with AS1411 aptamer not only improved the stability of the liposomes but also provided a supplementary steric barrier, resulting in the lower cumulative release of 5-FU. These liposomes demonstrated higher efficiency in terms of in vitro cell viability, apoptotic effects and targeting capability on human dermal fibroblasts and basal cell carcinoma TE 354 cell line than liposomes without functionalization [210].

In a different investigation, solid lipid NPs (liposomes) were developed for the adsorption of tumour-specific proteins in murine models of the subcutaneous melanoma model (B16-F10) and human lung carcinoma xenograft model (A549) that could not be detectable by plasma analysis. After the IV administration to tumour-bearing mice, the blood circulating liposomes captured and amplified a wide range of different proteins, including tumour-specific proteins (intracellular products of tissue leakage) called protein corona. Thus, the in vivo liposome protein corona could be an extraordinary tool to enrich blood proteomic analysis and help in the discovery of potential biomarkers for various disease models [211].

#### Niosomes

Niosomes are non-ionic surfactant-based vesicles that are developed to circumvent the stability issue of liposomes. Regarding cutaneous permeation for melanoma treatment, niosomes outperform liposomes in terms of drug entrapment efficiency and permeability due to non-ionic surfactants that change the stratum corneum integrity [212]. They have the additional advantages of low production cost, longer self-life, and the capability of controlling their bilayer fluidity and microviscosity [213].

Chermahini and Najaf [214] have developed 5FU-loaded niosomes for the treatment of basal cell skin cancer. The encapsulation of 5FU inside the niosomes not only improved the stability (with a zeta potential of 60) but also enhanced the skin permeation ability. To circumvent the poor percutaneous permeation, sulforaphane, a natural dietary isothiocyanate was loaded into ultradeformable vesicles such as ethosomes and transferosome. Out of the two types of vesicles, ethosomes with 2 % phospholipon 90G and 40 % ethanol showed higher stability than transferosomes. Drug-loaded ethosomes demonstrated enhanced percutaneous permeation of sulforaphane through the epidermis membrane and human stratum corneum. In addition, in vitro anticancer study on SK-MEL 28 cell lines revealed the ethosomes had improved anticancer activity due to the antiproliferative activity of sulforaphane [215].

#### Solid lipid nanoparticles

SLNs are colloidal particles prepared from physiological solid lipids (liquid at room temperature but solid at body temperature) such as tripalmitin, trimyristin, stearic acid, etc, surfactants (e.g., Poloxamer) employing most common techniques such as homogenization, ultrasonication and emulsification [216]. The potential advantages of SLNs are that protection of labile drugs from degradation, organic solvents-free preparation, higher drug loading, absence of toxicity due to the presence of physiological lipids, excellent tolerability, controlled release, can be scaled-up with reproducibility, and higher physical stability compared to liposomes [217,218]. In addition, the characteristics of SLNs are most favoured by hydrophobic drugs with high surface area and drug loading [219]. They also provide excellent opportunities for surface modification. For instance, surface modification with PEG molecules stops the opsonization by reticuloendothelial cells, thus improving circulation time and bioavailability [186]. However, they have issues with poor drug loading and leakage of drugs during storage.

The combination of drugs against the treatment of cancer is a bone as they provide a potential synergistic effect. However, it is limited by the absence of a suitable carrier that can deliver drugs precisely and helps to accumulate drugs in the cancerous cells. In this context, Cordeiro *et al.* [11] developed beeswax-based NPs simultaneously loaded with a hydrophobic drug, 4-nitrochalcone (4NC) and a hydrophilic drug, sodium diethyldithiocarbamate (DETC), for the treatment of melanoma cells (B16F10) synergistically. This nanosystem showed higher cytotoxicity towards melanoma cells compared to free drugs. In addition, the system was able to reduce the cell viability from 46 % for DETC-based NPs and 54 % for 4NC-based NPs to 64%. Moreover, the IC50 of the two drugs was found to be lower than that of individual drugs when encapsulated in beeswax NPs.

### Extracellular vesicles

#### Exosomes

Exosomes are one type of nano-sized (30-100nm in diameter) lipid-based bilayer vesicles [220,221], secreted by most living cells through the endocytosis process and responsible for cell-cell communications [222]. Primarily, exosomes are employed as biomarkers for diagnostics and potential natural drug delivery vehicles. As a drug carrier, exosomes have many advantages, such as the ability to penetrate across a variety of biological barriers, including blood-brain-barrier (BBB), natural targeting capability, biocompatible, less immunogenic compared to artificial nanocarriers, high stability and ability to deliver both large biological molecules such as genetic materials, various protein and lipids and small active drug molecules [223]. In addition, exosomes can take advantage of receptor-ligand interaction to enter into the cytosol, whereas synthetic lipid-based NPs have the tendency to accumulate on cell membranes [224]. After reaching the cytosol of the cell, exosomes fuse with the endosomes and deliver the loaded drugs. Thus, exosomes have the ability to improve drug delivery efficiency and targeting [225]. However, exosomes capacity to deliver a significant amount of active ingredients is restricted and the separation with high purity is labour-intensive and time-consuming. Thus, genetic engineering, followed by the cell culture method, has been adopted for the production of large amounts of exosomes. The drug loading into exosomes is a crucial step in drug delivery, which can be performed either endogenously or exogenously. For the endogenous approach, the exosomes are genetically modified to increase drug-containing exosome production, whereas the exogenous method involves loading drugs into them using encapsulation techniques such as sonication, electroporation, incubation and extrusion [226-228].

Most recently, Feng *et al.* [229] developed a coordination-driven assembly of Ir(III) photosensitizer with Fe(III) ions into nanopolymers (Ir-Fe NPs) for synergistic PDT and CDT therapy against melanoma. Further, these NPs were camouflaged with melanoma exosomes to form Ir-Fe@Exo to further their cancer selectivity and stability. Upon exposure to two-photon irradiation, Ir(III) complexes generate a combination of singlet oxygen and superoxide anion radicals that have the capability to induce cell death through autophagy and apoptosis. On the other hand, Fe(III) ions are involved in the depletion of glutathione, the generation of lipid peroxide and the lowering of GPX4 levels within the cancer cells, thereby the induction of ferroptosis. The obtained exosomes demonstrated enhanced blood circulation and high tumour accumulation inside a melanoma xenograft mouse model. In addition, melanoma tumours were fully eradicated with a single treatment of prepared exosomes intravenously followed by two-photon irradiation. Furthermore, these exosomes prevent the formation of lung metastasis inside the mouse model.

## Gene therapy for melanoma

The fast progress in science in the 21^st^ century made it realise that gene delivery for various genetic diseases is achievable. However, numerous challenges are involved when naked nucleic acids are subjected to systemic administration, including enzymatic degradation and non-specific delivery. Therefore, there is an urgent need to explore and develop smart nanocarriers that will be able to load the genetic material followed by their passage through various cellular barriers and release them into the cell nucleus or surrounding compartment successfully [230,231]. In this regard, two approaches, such as filomicelle and nanoworm iron-oxide particles, were successfully applied for the delivery of siRNA.

In one investigation, Wang *et al.* [232] employed a novel type of nanoworm, biomimetic nanoerythrocytes, for siRNA delivery for the treatment of melanoma. This approach involves red blood cell (RBC) membrane clocking of charge-reversible polyplexes of siRNA and polycation. Here, the clocking of the RBC membrane protects siRNA from degradation by the RNase enzyme. This RBC-reversible polyplex (RP) not only increased the blood circulation time compared to negatively and positively charged bovine serum albumin spheres but was also capable of escaping from late endosomes/lysosomes for the effective transfection of gene knockdown. Due to exceptional biocompatibility and active targeting (Cyclic Arg-Gly-Asp, cRGD), RGD-RBC-RP demonstrated a superior anti-melanoma effect in an animal model after two weeks of study.

Reported research on types of NMs, types of DDS along with the therapeutic molecules, polymers and the obtained results are summarized in Table 1.

**Table 1.** The summary of the reported research involving types of NMs and DDS, along with therapeutic molecules, polymers and the obtained result

Types of NM	Types of DDS	Therapeutic agent	Polymer	Result	Ref.
Gold NPs	Nanocomposite (Gold Nanoclusters)	AZD8055	Albumin	Significant cellular toxicity with decreasing the tumor surface	[80]
	Nanocomposite	Chalcones	-	Antitumor activity through the generation of ROS	[81]
	NPs	Paclitaxel	-	Antimelanoma activity through the generation of ROS and induction of apoptosis	[83]
	Nanocomposite (Gold nanorods)	-	Gum arabic	Reduced primary tumour growth by 45 % and reduced melanoma metastasis	[9]
	Gold-nanoshell	Doxorubicin	Antibody and PEG	Expression of significantly higher level of PD-L1. Precise targeting and delivery of drugs to melanoma cells	[84]
	Theranostic nanoprobe	-	p-MBA Anti-MUC18 single chain antibody (scFy)	In vivo melanoma site tracing, biodistribution study and targeted treatment	[86]
	Nanocomposite	-	HA and oleic acid	More effective against non-metastatic melanoma or other superficial tumours	[87]
	Nanocomposite	-	Folic acid	Decreased viability of melanoma cells and decrease in side effects.	[88]
Gold NPs and TiO_2_ nanorods	Nanocomposite	-	-	Reduction of cell viability to less than 1 %	[89]
Au-MnO_2_ nano-composite	Nanocomposite	-	-	Cytotoxicity of C540 cell lines in a dose-dependent manner	[90]
Silver NPs	Nanocomposite hydrogel		Chitosan and starch	A dose-dependent decrease in % of cell viability	[98]
	Organometallic Nanocomposite		Pheophorbide-a(Ph-a)	Effective suppression of tumour growth in vivo	[99]
	Nanohybrid bio-hydrogel		CMC and poly(acrylic acid-co-maleic acid	Reduced the viability of melanoma cells in a dose and time-dependent manner by the generation of ROS and cell death	[100]
	Nanohybrid hydrogel	Doxorubicin	CMC	Killing of melanoma cells synergistically and antibacterial effect	[101]
	Nanocomposite	Cisplatin	PVP	Induction of both apoptosis and necrosis	[102]
Silver NPs and platinum NPs	Nanocomposite	-	-	Selective cytotoxicity of melanoma cells through enhanced permeability and retention in these cells	[2]
CTNPs	Ferroptosis-activating vaccine	-	OVA and melanoma B16-OVA membrane	Trigger ferroptosis and induction of immunogenic cell death through strong immune response in vivo	[110]
CuO_2_NPs	Nanocomposite	-	PVP	Transform endogenous H_2_O_2_ into highly toxic OH and glutathione (GSH)-scavenging for ROS-induced tumour cell death	[21]
Pt-NPs	Nanocomposite	Doxorubicin	PEG	inhibition of cancer cell proliferation and induced apoptosis of cancer cells	[113]
Pt mesoporous NPs (Pt-MNPs)	NPs	-	---	Low melanoma cell viability (L1 %) due to significant generation of ROS	[119]
Silica NPs	Nanocomplex	Curcumin	-	Higher cancerous cell death due to the formation ROS	[125]
Mesoporous silica NPs	Nanocomplex	Verteporfin	-	Selectively reduced cancer cell proliferation	[28]
Mesoporous-silica NPs	NPs	-	-	Selective tumour growth inhibition due to higher amount ROS formation	[126]
PEGylated Silica NPs	Core-shell NPs	-	PEG and α_v_β3-integrin melanoma targeting peptides	Precise targeting and induced ferroptosis in starved cancer cells	[127]
Mesoporous silica NPs	2D core-shell nanosystem with silica layer	Cisplatin	-	Synergistic pyroptosis and hyperthermia of melanoma carcinoma	[129]
MCM	NPs	Quercetin	-	Improves stability of quercetin and enhanced quercetin penetration across skin	[130]
Gold NPs and mesoporous silica NPs	Gold nanoshell on rod-like mesoporous silica NPs	-	-	Effective penetration into melanoma tissues and potential ablation of malignant melanoma cells	[128]
TiO_2_ NPs	Nanocomposite	Curcumin	-	DNA damage, apoptosis and stopping the cells at the G2/M phase	[135]
Iron NPs	MPN nanocomposite	Cabazitaxel	Chitosan	Higher retention time in tumor tissues and thereby significant reduction of tumor growth	[144]
	MPN nanocomposite	Gallic acid	Hyaluronic acid	Synergistic therapy (PDT and CDT) against melanoma due to the generation of ROS	[145]
	Nanocomposite - hydrogel	Polydopamine-Fe(III)-doxorubicin NP	Alginate	Significant inhibition of proliferation and migration of melanoma cells due to hyperthermia. It also induces skin generation.	[146]
SWCNTs	Nanovector	Doxorubicin	-	Ceased the tumour growth without producing systemic side effects	[154]
MWCNTs	Nanocomposite	Polypyrrole	-	Concentration-dependent cytotoxicity with a cell viability of 8.9% due to hyperthermia	[155]
rGO	Nanocomposite hydrogel	5-fluorouracil	CMARX	Significant anticancer activities and antibacterial activities	[167]
nGO	Nanocomposite sheets	Dacarbazine	Chitosan oligosaccharide and CD47 antibody	Simultaneous induction of apoptosis through the mitochondria apoptosis pathway	[168]
Graphene	Nanotransporter	Fluorescence protein expression plasmid DNA (pDNA)	Magnetic NPs and PEI	Efficient delivery of pDNA into the melanoma cells through condensation	[169]
Nano-diamonds	NPs	-	-	Potential reduction of the metastasis through inhibition of the migration of Hela cells	[170]
Polymeric nanocapsules	NPs	Curcumin	PBA and AEMA	Selectively killed more than 90% of human malignant melanoma cells	[183]
	Hybrid NPs	Vitamin D3	PLGA and hydrogenated soy phosphatidylcholine, cholesterol and 1,2-disteroyl-sn-glycero-3-phospha ethanolamine-N[succinyl (PEG)-2000	Target B16 melanoma cells efficiently	[184]
	NPs	Celecoxib and afuresertib	PLGA, PEG and targeting peptide IL4RPep-1	Enhancement of apoptosis and regulation of M2 macrophages in tumor cells to reprogram M1 macrophages	[185]
Dendrimers	Nanoconjugates	Temozolomide	PAMAM-PEG-GE11-HA	Targeting towards A375 human melanoma cell	[189]
	Core-shell nanomicelles	Dabrafenib and vemurafenib	G2 PAMAM head and C18 aliphatic chains	Release of drugs enhanced at acidic pH 5.0 and enhanced antimelanoma response	[7]
Polymeric micelles	Nanomicelleles	Doxorubicin	mPEG-b-PLAGA and IR-768	Triggered synergistic anticancer effect in melanoma cells	[192]
Nanogel	Nanogel	GOX	FBMA and OEGMA (poly(FBMA-co-OEGMA)	Improved antimelanoma efficacy without systemic toxicity due to the confinement of GOX to only tumour region	[197]
	Thermosensitive nano-hydrogel	PP-NPs containing deferasirox	Agarose and TMPO-oxidized lignocellulose	Dose-dependent cytotoxicity	[198]
Liposomes	Liposomes + Vaccines	Celecoxib	Dendritic cell vaccines pulsed with gp100 peptide	Tumour growth inhibition and overall survival	[207]
	Chitosan coated liposomes	Indocyanine green	Chitosan	Higher cellular uptake and photocytotoxicity of melanoma cells due to high skin permeability	[208]
	HA-coated liposomes	Dacarbazine and eugenol	HA	Higher cytotoxicity against melanoma cells along with significantly higher inhibition of cell migration and proliferation	[209]
	AS1411 aptamer functionalized liposomes	5-fluorouracil	AS1411 aptamer	Higher efficiency in terms of in vitro cell viability, apoptotic effects and targeting capability	[210]
	-	-	-	Tool to enrich the blood proteomic analysis	[211]
Niosomes	-	5-fluorouracil	-	Improved the stability and enhanced the skin permeation ability	[214]
Ethosomes and transferosome	-	Sulforaphane	-	Higher skin permeability and improved anticancer activity due to antiproliferative activity	[215]
SLNs	-	4NC and DETC	Beeswax	Higher cytotoxicity reduces cell viability	[11]
Exosomes	Ir-Fe@Exo	-	Ir-Fe NPs camouflage with melanoma exosomes	Full eradication of melanoma tumour with single treatment and prevent the metastasis	[229]
Gene therapy	Nanoworm	siRNA (siSuvivin)	Nanoerythrocytes	Protects siRNA and superior anti-melanoma effect	[232]

## Conclusions

The last few decades have witnessed extraordinary progress in the development of materials that are oriented towards melanoma therapy. The most important benefit of NMs is that they can not only be directly delivered to treat melanoma but also can carry and deliver other therapeutic agents to achieve better or synergistic therapeutic effects. NM-based DDS or nanomedicine has opened new avenues in the therapeutic management of complex diseases such as melanoma skin cancer. Recently, successful and encouraging results from the preclinical and clinical investigations brought hope in the anticipated direction. Despite the fact that nanotherapeutics have come a long way in significant anti-melanoma therapy, undoubtedly, there exist a few key issues, such as the requirement of high initial investment, scalability, safety (toxicity), purity, lack of proper guidelines (nano guidelines), and more importantly lack of good in-vitro and pre-clinical melanoma models.

The main starting issue for pharmaceutical companies in the development of nanomedicine for melanoma is the high capital investment coupled with the fear of failure in the return of investment [233]. The batch-to-batch variation after manufacturing of nanomedicine is critical due to the complex nature of quality control they undergo compared to conventional dosage forms. The transition from laboratory scale to industrial scale is also challenging because of the requirement of a lot of effort per stability and optimization. However, the invention of the microfluidic technique could streamline the scalability issue [234]. The safety of the NMs is also very crucial during the development of nanotherapeutics. Some inorganic NMs demonstrated long-term toxicities and issues related to biodegradability. It is evident from the cytotoxicity caused by dendrimer. Apart from NMs, the excipients used in the preparation of nanotherapeutics, including emulsifiers, solvents like alcohol, and edge activators, have the potential to cause skin irritation and toxicity. The regulatory landscapes for NM-based DDS are still in the infancy stage, necessitating urgent reform. In this context, it can be more appropriate to develop ‘nanoguidelines” that will erase the uncertainty among investors and the pharma industry [233]. For the successful launch of nanomedicines, proper in-vitro, pre-clinical and clinical models are desired. For example, human-specific and physiologically relevant models such as 3D spheroids-based models and human skin equivalent models are better predictors of in-vivo models. Despite all these, it is believed that with the continuous development in nanotechnology and NM-based DDS, the challenges will be addressed in the foreseeable future, which will pave the path for the better management of dreaded diseases such as skin melanoma.

## Abbreviations

DDS: Drug delivery system
UV: Ultraviolet
BRAF: v-Raf murine sarcoma viral oncogene homolog B1
NRAS: Neuroblastoma RAS viral oncogene homolog
NFI: Nuclear factor I
CTLA-4: Cytotoxic T-lymphocyte–associated antigen 4
PD-1: Programmed death 1
PDT: Photodynamic therapy
PTT: Photothermal therapy
NPs: Nanoparticles
NMs: Nanomaterials
SLN: Solid lipid nanoparticles
mAb: Monoclonal antibodies
RGP: Radial growth phase
VGP: Vertical growth phase
CDKN2A: Cyclin-dependent kinase inhibitor 2A
MAPK: Mitogen-activated protein kinase
P13K/AKT: Phosphatidylinositol 3-kinase
c-KIT: Tyrosine kinase receptor
TILs: Tumour infiltration lymphocytes
PD-L1: Programmed death ligand-1
Tregs: Regulatory T cells
MDSCs: Myeloid-derived suppressor cells
ROS: Reactive oxygen species
LEDs: Light-emitting diodes
PEGylation: Polyethylene glycol conjugation
EPR: Enhanced permeability and retention
mTOR: Mammalian target of rapamycin
SDT: Sonodynamic therapy
HA: Hyaluronic acid
p-MBA: 4-mercapto benzoic acid
CMC: Carboxymethyl cellulose
ODA: Octadecylamine
CDT: Chemodynamic therapy
Cu2O-NPs: Cuprous oxide NPs
CTNPs: Copper telluride NPs
OVA: Ovalbumin
UCNs: Upconversion fluorescent NPs
MCM-41: Mobil composition of matter no 41
MPN: Metal-polyphenol network
Ce6: Chlorin e6
SD: Alginate-graft-dopamine
PFD: Polydopamine-Fe(III)-doxorubicin NPs
CNTs: Carbon nanotubes
SWCNTs: Single-walled CNTs
MWCNTs: Multi-walled CNTs
PP: Polypyrrole
GO: Graphene oxide
rGO: Reduced GO
CMARX: Carboxymethylarabinoxylan
pDNA: Plasmid DNA
GEMNs: Graphene-encapsulated magnetic NPs
PEI: Polyethyleneimine
TGF-β: Factor β signalling pathway
PLA: Poly(lactic acid)
PGA: Poly (glycolic acid)
PLGA: Poly(lactic-co-glycolic acid)
PCL: Poly(P-caprolactone)
PBA: Phenylboronic acid
AEMA: 2-aminoethylmethacrylate
PAMAM: Polyamide amine dendrimer
GOX: Glucose oxidase
FBMA: 4-formyl-N-(3-(2-(2-(3-methacrylamidopropoxy)ethoxy)-ethoxy)propyl) benzamide
OEGMA: Oligo ethylene glycolmonomethyl ether methacrylate
PC: Phosphatidylcholine
PE: Phosphatidylethanolamine
4NC: 4-nitrochalcone
DETC: Sodium diethyldithiocarbamate
BBB: Blood-brain-barrier
RBC: Red blood cell

## References

[1] Bromma K. Cicon L. Beckham W. Chithrani D.B.. Gold nanoparticle mediated radiation response among key cell components of the tumour microenvironment for the advancement of cancer nanotechnology. Scientific Reports 10 (2020) 12096. https://www.nature.com/articles/s41598-020-68994-0. 32694592 10.1038/s41598-020-68994-0 PMC7374632

[2] Ruiz A.L. Garcia C.B. Gallón S.N. Webster T.J.. Novel silver-platinum nanoparticles for anticancer and antimicrobial applications. International Journal of Nanomedicine 15 (2020) 169-179. https://doi.org/10.2147/IJN.S176737. 10.2147/IJN.S176737 32021172 PMC6970512

[3] Wong X.Y. Sena-torralba A. Álvarez-Diduk R. Muthoosamy K. Merkoc A.. Nanomaterials for nanotheranostics: Their Properties According to Disease Needs. ACS Nano 14 (2020) 2585-2627. https://doi.org/10.1021/acsnano.9b08133. 10.1021/acsnano.9b08133 32031781

[4] Navya P.N. Kaphle A. Srinivas S.P. Bhargava S.K. Rotello V.M. Daima H.K.. Current trends and challenges in cancer management and therapy using designer nanomaterials. Nano Convergence 6 (2019) 23. https://doi.org/10.1186/s40580-019-0193-2. 10.1186/s40580-019-0193-2 31304563 PMC6626766

[5] Das C.G.A. Kumar V. Dhas T.S. Karthick V. Kumar C.M.V.. Nanomaterials in anticancer applications and their mechanism of action- A review. Nanomedicine: Nanotechnology, Biology, and Medicine 47 (2023) 102613. https://doi.org/10.1016/j.nano.2022.102613. 10.1016/j.nano.2022.102613 36252911

[6] Arnold M. Singh D. Laversanne M. Vignat J. Vaccarella S. Meheus F. Cust A.E. de Vries E. Whiteman D.C. Bray F.. Global burden of cutaneous melanoma in 2020 and projections to 2040. JAMA Dermatology 158 (2022) 495-503. https://doi.org/10.1001/jamadermatol.2022.0160. 10.1001/jamadermatol.2022.0160 35353115 PMC8968696

[7] Russi M. Valeri R. Marson D. Danielli C. Felluga F. Tintaru A. Skoko N. Aulic S. Laurini E. Pricl S.. Some things old, new and borrowed: Delivery of dabrafenib and vemurafenib to melanoma cells via self-assembled nanomicelles based on an amphiphilic dendrimer. European Journal of Pharmaceutical Sciences 180 (2023) 106311. https://doi.org/10.1016/j.ejps.2022.106311. 10.1016/j.ejps.2022.106311 36273785

[8] Bray F. Ferlay J. Soerjomataram I. Siegel R.L. Torre L.A. Jemal A.. Global Cancer Statistics 2018: GLOBOCAN Estimates of Incidence and Mortality Worldwide for 36 Cancers in 185 Countries. CA: A Cancer Journal for Clinicians 68 (2018) 394-424. https://doi.org/10.3322/caac.21492. 10.3322/caac.21492 30207593

[9] Gonçalves J.P. da Cruz A.F. Nunes A.M. Meneghetti M.R. de Barros H.R. Borges B.S. de Medeiros L.C.A.S. Soares M.J. dos Santos M.P. Grassi M.T. Rossi G.R. Bellan D.L. Biscaia S.M.P. Cristal A.M. Buzzo J.L.A. Ribeiro Y.C. Acco A. Cardoso M.B. Simas F.F. Trindade E.S. Riegel-Vidotti I.C. de Oliveira C.C.. Biocompatible gum arabic-gold nanorod composite as an effective therapy for mistreated melanomas. International Journal of Biological Macromolecules 185 (2021) 551-561. https://doi.org/10.1016/j.ijbiomac.2021.06.172. 10.1016/j.ijbiomac.2021.06.172 34216657

[10] Cassano R. Cuconato M. Calviello G. Serini S. Trombino S.. Recent Advances in Nanotechnology for the Treatment of Melanoma. Molecules 26 (2021) 785. https://doi.org/10.3390/molecules26040785. 10.3390/molecules26040785 33546290 PMC7913377

[11] Cordeiro A.P. Feuser P.E. Figueiredo P.G. da Cunha E.S. Martinez G.R. Machado-de-Avila R.A. Rocha M.E.M. de Araújo P.H.H. Sayer C.. In vitro synergic activity of diethyldithiocarbamate and 4-nitrochalcone loaded in beeswax nanoparticles against melanoma (B16F10) cells. Materials Science & Engineering C 120 (2021) 111651. https://doi.org/10.1016/j.msec.2020.111651. 10.1016/j.msec.2020.111651 33545819

[12] Liu Q. Das M. Liu Y. Huang L.. Targeted drug delivery to melanoma. Advanced Drug Delivery Reviews 127 (2018) 208-221. https://doi.org/10.1016/j.addr.2017.09.016. 10.1016/j.addr.2017.09.016 28939379

[13] DeSantis C.E. Lin C.C. Mariotto A.B. Siegel R.L. Stein K.D. Kramer J.L. Alteri R. Robbins A.S. Jemal A.. Cancer treatment and survivorship statistics, 2014. CA. A Cancer Journal for Clinicians 64 (2014) 252-271. https://doi.org/10.3322/caac.21235. 10.3322/caac.21235 24890451

[14] Welsh S.J. Corrie P.G.. Management of BRAF and MEK inhibitor toxicities in patients with metastatic melanoma. Therapeutic Advances in Medical Oncology 7 (2015) 122-136. https://doi.org/10.1177/1758834014566428. 10.1177/1758834014566428 25755684 PMC4346212

[15] Blankenstein S.A. van Akkooi A.C.J.. Adjuvant systemic therapy in high-risk melanoma. Melanoma Research 29 (2019) 358-364. https://doi.org/10.1097/CMR.0000000000000604. 10.1097/CMR.0000000000000604 30896556

[16] National Cancer Institute, Drugs approved for skin cancer, https://www.cancer.gov/about-cancer/treatment/drugs/skin. August 16, 2023.

[17] Berk-Krauss J. Stein J.A. Weber J. Polsky D. Geller A.C., New systematic therapies and trends in cutaneous melanoma deaths among US whites, 1986-2016. American Journal of Public Health 110 (2020) 731-733. https://doi.org/10.2105/AJPH.2020.305567. 10.2105/AJPH.2020.305567 32191523 PMC7144422

[18] Payandeh Z. Yarahmadi M. Nariman-Saleh-Fam Z. Tarhriz V. Islami M. Aghdam A.M. Eyvazi S.. Immune therapy of melanoma: overview of therapeutic vaccines. Journal of Cellular Physiology 234 (2019) 14612-14621. https://doi.org/10.1002/jcp.28181. 10.1002/jcp.28181 30706472

[19] Zhao B. He Y.-Y.. Recent advances in the prevention and treatment of skin cancer using photodynamic therapy. Expert Review of Anticancer Therapy 10 (2010) 1797-1809. https://doi.org/10.1586/era.10.154. 10.1586/era.10.154 21080805 PMC3030451

[20] Zeng Y. Chen J. Tian Z. Zhu M. Zhu Y.. Preparation of mesoporous organosilica based nanosystem for in vitro synergistic chemo- and photothermal therapy. Journal of Inorganic Materials 35 (2020) 1365-1372. https://doi.org/10.15541/jim20200091. 10.15541/jim20200091

[21] Chen J. Cao Y. Lin S. Niu H. Zhang H. Guan L. Shu C. Wu A. Bian Y. Zhu Y.. A responsive microneedle system for efficient anti-melanoma by combining self-enhanced chemodynamic therapy with photothermal therapy. Chemical Engineering Journal 431 (2022) 133466. https://doi.org/10.1016/j.cej.2021.133466. 10.1016/j.cej.2021.133466

[22] Sun T. Zhang Y.S. Pang B. Hyun D.C. Yang M. Xia Y., Engineered nanoparticles for drug delivery in cancer therapy, Angewandte Chemie 53 (2014) 12320-12364. https://doi.org/10.1002/anie.201403036. 10.1002/anie.201403036 25294565

[23] Wilczewska A.Z. Niemirowicz K. Markiewicz K.H. Car H., Nanoparticles as drug delivery systems, Pharmacol. Rep. 64 (2012) 1020-1037. https://doi.org/10.1016/S1734-1140(12)70901-5. 10.1016/S1734-1140(12)70901-5 23238461

[24] Singh R. Lillard J.W. Jr. Nanoparticle-based targeted drug delivery. Experimental and Molecular Pathology 86 (2009) 215-223. https://doi.org/10.1016/j.yexmp.2008.12.004. 10.1016/j.yexmp.2008.12.004 19186176 PMC3249419

[25] Pourmadadi M. Mahdi Eshaghi Md. Ostovar S. Mohammadi Z. Sharma R.K. Paiva-Santos A.C. Rahmani E. Rahdar A. Pandey S.. Innovative nanomaterials for cancer diagnosis, imaging, and therapy: Drug delivery applications. Journal of Drug Delivery Science and Technology 82 (2023) 104357. https://doi.org/10.1016/j.jddst.2023.104357. 10.1016/j.jddst.2023.104357

[26] Liu T. Lu Y. Zhan R. Qian W. Luo G.. Nanomaterials and nanomaterials-based drug delivery to promote cutaneous wound healing. Advanced Drug Delivery Reviews 193 (2023) 114670. https://doi.org/10.3390/nano12040618. 10.3390/nano12040618 36538990

[27] Alarifi S. Ali D. Alkahtani S. Verma A. Ahamed M. Ahmed M. Alhadlaq H.A.. Induction of oxidative stress, DNA damage, and apoptosis in a malignant human skin melanoma cell line after exposure to zinc oxide nanoparticles. International Journal of Nanomedicine 8 (2013) 983. https://doi.org/10.2147/ijn.s42028. 10.2147/ijn.s42028 23493450 PMC3593769

[28] Rizzi M. Tonello S. Esteva˜o B.M. Gianotti E. Marchese L. Reno F.. Verteporfin based silica nanoparticle for in vitro selective inhibition of human highly invasive melanoma cell proliferation. Journal of Photochemistry and Photobiology B. 167 (2017) 1-6. https://doi.org/10.1016/j.jphotobiol.2016.12.021. 10.1016/j.jphotobiol.2016.12.021 28039784

[29] Zielinska A. Pereira I. Antunes S. Veiga F.J. Santos A.C. Nowak1 I. Silva A.M. Souto E.B.. Mesoporous silica nanoparticles as drug delivery systems against melanoma, In Design of Nanostructures for Theranostics Applications, Grumezescu A.M., (Ed.)., Elsevier Inc., 2018, p. 437-466. http://dx.doi.org/10.1016/B978-0-12-813669-0.00010-5. 10.1016/B978-0-12-813669-0.00010-5

[30] Grosu-Bularda A. Lazarescu L. Stoian A. Lascar I.. Immunology and skin cancer. Archives of Clinical Cases 5 (2018) 109-119. https://doi.org/10.22551/2018.20.0503.10137. 10.22551/2018.20.0503.10137

[31] Katalinic A. Kunze U. Schafer T.. Epidemiology of cutaneous melanoma and nonmelanoma skin cancer in Schleswig-Holstein, Germany: incidence, clinical subtypes, tumour stages and localization (epidemiology of skin cancer). Brazilian Journal of Dermatology. 149 (2003) 1200-1206. https://doi.org/10.1111/j.1365-2133.2003.05554.x. 10.1111/j.1365-2133.2003.05554.x 14674897

[32] Gloster H.M. Jr. Brodland D.G.. The epidemiology of skin cancer. Dermatologic Surgery 22 (1996) 217-226. https://doi.org/10.1111/j.1524-4725.1996.tb00312.x. 10.1111/j.1524-4725.1996.tb00312.x 8599733

[33] Bradford P.T.. Skin cancer in skin of color. Dermatology Nursing 21 (2009) 170-177 (206; quiz 178). https://pubmed.ncbi.nlm.nih.gov/19691228/. 19691228 PMC2757062

[34] Leiter U. Keim U. Garbe C.. Epidemiology of Skin Cancer: Update 2019, in Sunlight, Vitamin D Skin Cancer, Reichrath J. (Ed.)., Springer, Cham., 2020, p. 123-139. https://doi.org/10.1007/978-3-030-46227-7-6. 10.1007/978-3-030-46227-7-6 32918216

[35] Hussein M.R.. Ultraviolet radiation and skin cancer: molecular mechanisms. Journal of Cutaneous Pathology 32 (2005) 191-205. https://doi.org/10.1111/j.0303-6987.2005.00281.x. 10.1111/j.0303-6987.2005.00281.x 15701081

[36] A.I.M. foundation, About Melanoma https://www.aimatmelanoma.org/stages-of-melanoma/. September 9, 2023

[37] Rastrelli M. Tropea S. Rossi C.R. Alaibac M., Melanoma: epidemiology, risk factors, pathogenesis, diagnosis and classification. In Vivo 28 (2014) 1005-1011. https://iv.iiarjournals.org/content/28/6/1005. 25398793

[38] Cummins D.L. Cummins J.M. Pantle H. Silverman M.A. Leonard A.L. Chanmugam A.. Cutaneous malignant melanoma. Mayo Clinic Proceedings 81 (2006) 500-507. https://doi.org/10.4065/81.4.500. 10.4065/81.4.500 16610570

[39] Carr S. Smith C. Wernberg J.. Epidemiology and risk factors of melanoma. Surgical Clinics 100 (2020) 1-12, https://doi.org/10.1016/j.suc.2019.09.005. 10.1016/j.suc.2019.09.005 31753105

[40] Li J. Zhang Y. Tao J.. Targeted Nanoparticles for Drug Delivery to Melanoma: From Bench to Bedside. In Nanoscience in Dermatology, Hamblin M.R. Avci P. Prow T.W., (Eds.)., Elsevier Inc., p. 203-215. http://dx.doi.org/10.1016/B978-0-12-802926-8.00016-1. 10.1016/B978-0-12-802926-8.00016-1

[41] Hodis E. Watson I.R. Kryukov G.V. Arold S.T. Imielinski M. Theurillat J.P. Nickerson E. Auclair D. Li L. Place C. Dicara D. Ramos A.H. Lawrence M.S. Cibulskis K. Sivachenko A. Voet D. Saksena G. Stransky N. Onofrio R.C. Winckler W. Ardlie K. Wagle N. Wargo J. Chong K. Morton D.L. Stemke-Hale K. Chen G. Noble M. Meyerson M. Ladbury J.E. Davies M.A. Gershenwald J.E. Wagner S.N. Hoon D.S.B. Schadendorf D. Lander E.S. Gabriel S.B. Getz G. Garraway L.A. Chin L.. A landscape of driver mutations in melanoma. Cell 150 (2012) 251-163. https://doi.org/10.1016/j.cell.2012.06.024. 10.1016/j.cell.2012.06.024 22817889 PMC3600117

[42] Vukovi´c P. Lugovi´c-Mihi´c L. Cesic D. Novak-Bili´c G. Situm M. Spoljar S., Melanoma development: current knowledge on melanoma pathogenesis. Acta Dermatovenerologica Croatica 28 (2020) 163-163. https://hrcak.srce.hr/225444. 31542060

[43] Prajapat V.M. Mahajan S. Paul P.G. Aalhate M. Mehandole A. Madan J. Dua K. Chellappan D.K. Singh S.K. Singh P.K.. Nanomedicine: A pragmatic approach for tackling melanoma skin cancer. Journal of Drug Delivery Science and Technology 83 (2023) 104394. https://doi.org/10.1016/j.jddst.2023.104394. 10.1016/j.jddst.2023.104394

[44] Blank C. Brown I. Peterson A.C. Spiotto M. Iwai Y. Honjo T. Gajewski T.F.. PD-L1/B7H-1 inhibits the effector phase of tumor rejection by T cell receptor (TCR) transgenic CD8þ T cells. Cancer Research 64 (2004) 1140-1145. https://doi.org/10.1158/0008-5472.can-03-3259. 10.1158/0008-5472.can-03-3259 14871849

[45] Mellman Coukos G. Dranoff G.. Cancer immunotherapy comes of age. Nature 480 (2011) (7378) 480-489. https://doi.org/10.1038/nature10673. 10.1038/nature10673 22193102 PMC3967235

[46] Garbe C. Eigentler T.K. Keilholz U. Hauschild A. Kirkwood J.M.. Systematic review of medical treatment in melanoma: current status and future prospects. Oncologist 16 (2011) 5-24. https://doi.org/10.1634/theoncologist.2010-0190. 10.1634/theoncologist.2010-0190 21212434 PMC3228046

[47] Das P. Deshmukh N. Badore N. Ghulaxe C. Patel P.. A review article on melanoma. Journal of Pharmaceutical Sciences and Research 8 (2016) 112-117. https://www.jpsr.pharmainfo.in/Documents/Volumes/vol8Issue02/jpsr08021610.

[48] Balch C.M. Gershenwald J.E. Soong S.J. Thompson J.F. Atkins M.B. Byrd D.R. Buzaid A.C. Cochran A.J. Coit D.G. Ding S. Eggermont A.M. Flaherty K.T. Gimotty P.A. Kirkwood J.M. McMasters K.M. Mihm M.C. Jr Morton D.L. Ross M.I. Sober A.J. Sondak V.K.. Final version of 2009 AJCC melanoma staging and classification. Journal of Clinical Oncology 27 (2009) 6199-6206. https://doi.org/10.1200/JCO.2009.23.4799. 10.1200/JCO.2009.23.4799 19917835 PMC2793035

[49] Phan G.Q. Attia P. Steinberg S.M. White D.E. Rosenberg S.A.. Factors associated with response to high-dose interleukin-2 in patients with metastatic melanoma. Journal of Clinical Oncology 19 (2001) 3477-3782. https://doi.org/10.1200/JCO.2001.19.15.3477. 10.1200/JCO.2001.19.15.3477 11481353

[50] Beiu C. Giurcaneanu C. Grumezescu A.M. Holban A.M. Popa L.G. Mihai M.M., Nanosystems for improved targeted therapies in melanoma. Journal of Clinical Medicine 9 (2020) 318. https://doi.org/10.3390/jcm9020318. 10.3390/jcm9020318 31979325 PMC7073828

[51] Smetana K. Jr. Lacina L. Kodet O., Targeted therapies for melanoma. Cancers 12 (2020) 2494. https://doi.org/10.3390/cancers12092494. 10.3390/cancers12092494 32899184 PMC7565572

[52] Ascierto P.A. Flaherty K. Goff S.. Emerging strategies in systemic therapy for the treatment of melanoma. American Society of Clinical Oncology Educational 38 (2018) 751-758. https://doi.org/10.1200/EDBK_199047. 10.1200/EDBK_199047 30231371

[53] Rigon R.B. Oyafuso M.H. Fujimura A.T. Gonçalez M.L. Prado A.H.d. Gremiao M.P.D. Chorilli M.. Nanotechnology-based drug delivery systems for melanoma antitumoral therapy. BioMed Research International 2015 (2015) 841817. https://doi.org/10.1155/2015/841817. 10.1155/2015/841817 26078967 PMC4442269

[54] Weinmann S.C. Pisetsky D.S.. Mechanisms of immune-related adverse events during the treatment of cancer with immune checkpoint inhibitors. Rheumatology 58(Suppl 7) (2019) vii59-vii67. https://doi.org/10.1093/rheumatology/kez308. 10.1093/rheumatology/kez308 31816080 PMC6900913

[55] Robert C. Ribas A. Wolchok J.D. Hodi F.S. Hamid O. Kefford R. Weber J.S. Joshua A.M. Hwu W.-J. Gangadhar T.C. Patnaik A. Dronca R. Zarour H. Joseph R.W. Boasberg P. Chmielowski B. Mateus C. Postow M.A. Gergich K. Elassaiss-Schaap J. Li X.N. Iannone R. Ebbinghaus S.W. Kang S.P. Daud A.. Antiprogrammed-death-receptor-1 treatment with pembrolizumab in ipilimumab-refractory advanced melanoma: a randomised dose-comparison cohort of a phase 1 trial. Lancet 384 (2014) 1109-1117. https://doi.org/10.1016/S0140-6736(14)60958-2. 10.1016/S0140-6736(14)60958-2 25034862

[56] Topalian S.L. Sznol M. McDermott D.F. Kluger H.M. Carvajal R.D. Sharfman W.H. Brahmer J.R. Lawrence D.P. Atkins M.B. Powderly J.D. Leming P.D. Lipson E.J. Puzanov I. Smith D.C. Taube J.M. Wigginton J.M. Kollia G.D. Gupta A. Pardoll D.M. Sosman J.A. Hodi F.S.. Survival, durable tumor remission, and long-term safety in patients with advanced melanoma receiving nivolumab. Journal of Clinical Oncology 32 (2014) 1020-1030. https://doi.org/10.1200/JCO.2013.53.0105. 10.1200/JCO.2013.53.0105 24590637 PMC4811023

[57] Tran M.A. Gowda R. Sharma A. Park E.-J. Adair J. Kester M. Smith N.B. Robertson G.P.. Targeting V600EB-Raf and Akt3 using nanoliposomal-small interfering RNA inhibits cutaneous melanocytic lesion development. Cancer Research 68 (2008) 7638-7649. https://doi.org/10.1158/0008-5472.CAN-07-6614. 10.1158/0008-5472.CAN-07-6614 18794153 PMC3628540

[58] Dasari S. Yedjou C.G. Brodell R.T. Cruse A.R. Tchounwou P.B., Therapeutic strategies and potential implications of silver nanoparticles in the management of skin cancer. Nanotechnology Reviews 9 (2020) 1500-1521. https://doi.org/10.1515/ntrev-2020-0117. 10.1515/ntrev-2020-0117 33912377 PMC8078871

[59] Zhao J. Gao N. Xu J. Zhu X. Ling G. Zhang P.. Novel strategies in melanoma treatment using silver nanoparticles. Cancer Letters 561 (2023) 216148. https://doi.org/10.1016/j.canlet.2023.216148. 10.1016/j.canlet.2023.216148 36990267

[60] Kim D. Amatya R. Hwang S. Lee S. Min K.A. Shin M.C.. BSA-silver nanoparticles: a potential multimodal therapeutics for conventional and photothermal treatment of skin cancer. Pharmaceutics 13 (2021) 575. https://doi.org/10.3390/pharmaceutics13040575. 10.3390/pharmaceutics13040575 33920666 PMC8073043

[61] Park T. Lee S. Amatya R. Cheong H. Moon C. Kwak H.D. Min K.A. Shin M.C.. ICG-loaded PEGylated BSA-silver nanoparticles for effective photothermal cancer therapy. International Journal of Nanomedicine 15 (2020) 5459-5471. https://doi.org/10.2147/IJN.S255874. 10.2147/IJN.S255874 32801700 PMC7406329

[62] Kasprzak J.M. Xu Y.G.. Diagnosis and management of lentigo maligna: a review. Drugs Context 4 (2015) 212281. https://doi.org/10.7573/2Fdic.212281. 10.7573/2Fdic.212281 26082796 PMC4453766

[63] Parhi R.. Recent advances in 3D printed microneedles and their skin delivery application in the treatment of various diseases. Journal of Drug Delivery Science and Technology 84 (2023) 104395. https://doi.org/10.1016/j.jddst.2023.104395. 10.1016/j.jddst.2023.104395

[64] Li X. Zhao Z. Zhang M. Ling G. Zhang P., Research progress of microneedles in the treatment of melanoma. Journal of Controlled Release 348 (2022) 631-647. https://doi.org/10.1016/j.jconrel.2022.06.021. 10.1016/j.jconrel.2022.06.021 35718209

[65] Bomar L. Senithilnathan A. Ahn C.. Systemic therapies for advanced melanoma. Dermatologic Clinics 37 (2019) 409-423. https://doi.org/10.1016/j.det.2019.05.001. 10.1016/j.det.2019.05.001 31466582

[66] Pecot C.V. Calin G.A. Coleman R.L. Lopez-Berestein G. Sood A.K., RNA interference in the clinic: challenges and future directions. Nature Reviews Cancer 11 (2011) 59-67. https://doi.org/10.1038/nrc2966. 10.1038/nrc2966 21160526 PMC3199132

[67] Khorsandi Z. Borjian-Boroujeni M. Yekani R. Varma R.S.. Carbon nanomaterials with chitosan: A winning combination for drug delivery systems. Journal of Drug Delivery Science and Technology 66 (2021) 102847. https://doi.org/10.1016/j.jddst.2021.102847. 10.1016/j.jddst.2021.102847

[68] Gong W. Zhang T. Che M. Wang Y. He C. Liu L. Lv Z. Xiao C. Wang H. Zhang S.. Recent advances in nanomaterials for the treatment of spinal cord injury. Materials Today Bio 18 (2023) 100524. https://doi.org/10.1016/j.mtbio.2022.100524. 10.1016/j.mtbio.2022.100524 PMC9813796 36619202

[69] El-Kady M.M. Ansari I. Arora C. Rai N. Soni S. Verma D.K. Singh P. El Din Mahmoud A.. Nanomaterials: A comprehensive review of applications, toxicity, impact, and fate to environment. Journal of Molecular Liquids 370 (2023) 121046. https://doi.org/10.1016/j.molliq.2022.121046. 10.1016/j.molliq.2022.121046

[70] Seaberg J. Clegg J.R. Bhattacharya R. Mukherjee P.. Self-therapeutic nanomaterials: Applications in biology and medicine. Materials Today 62 (2023) 190-224. https://doi.org/10.1016/j.mattod.2022.11.007. 10.1016/j.mattod.2022.11.007 36938366 PMC10022599

[71] Liong M. Lu J. Kovochich M. Xia T. Ruehm S.G. Nel A.E. Tamanoi F. Zink J.I.. Multifunctional inorganic nanoparticles for imaging, targeting, and drug delivery. ACS Nano 2 (2008) 889-896. https://doi.org/10.1021/nn800072t. 10.1021/nn800072t 19206485 PMC2751731

[72] Hassan S. Prakash G. Ozturk A.B. Saghazadeh S. Sohail Md.F. Seo J. Dokmeci M.R. Zhang Y.S. Khademhosseini A.. Evolution and clinical translation of drug delivery nanomaterials. Nano Today 15 (2017) 91-106. https://doi.org/10.1016/j.nantod.2017.06.008. 10.1016/j.nantod.2017.06.008 29225665 PMC5720147

[73] Faraday M.. X. The Bakerian Lecture-experimental relations of gold (and other metals) to light, Philosophical Transactions. Royal Society, London, 147 (1997) 145-181. https://doi.org/10.1098/rstl.1857.0011. 10.1098/rstl.1857.0011

[74] Thakor A.S. Jokerst J. Zavaleta C. Massoud T.F. Gambhir S.S.. Gold nanoparticles: a revival in precious metal administration to patients. Nano Letters 11 (2011) 4029-4036. https://doi.org/10.1021/nl202559p. 10.1021/nl202559p 21846107 PMC3195547

[75] Qu Y. Lian S. Shen W. Li Z. Yang J. Zhang H.. Rod-shaped gold nanoparticles biosynthesized using Pb2+-induced fungus Aspergillus sp.WLAu. Bioprocess and Biosystems Engineering 43 (2020) 123-131. https://doi.org/10.1007/s00449-019-02210-w. 10.1007/s00449-019-02210-w 31628532

[76] Dreaden E.C. Austin L.A. Mackey M.A. El-Sayed M.A.. Size matters: gold nanoparticles in targeted cancer drug delivery. Therapeutic Delivery. 3 (2012) 457-478. https://doi.org/10.4155/tde.12.21. 10.4155/tde.12.21 22834077 PMC3596176

[77] Jeong Y. Kim G. Jeong S. Lee B. Kim S. Koh W.-G. Lee K.. Cancer selective turn-on fluorescence imaging using a biopolymeric nanocarrier. Biomacromolecules 20(Span 80) (2019) 1068-1076. https://doi.org/10.1021/acs.biomac.8b01690. 10.1021/acs.biomac.8b01690 30645935

[78] Vemuri S.K. Banala R.R. Mukherjee S. Uppula P. Subbaiah G.P.V. Reddy A.V.G. Malarvilli T.. Novel biosynthesized gold nanoparticles as anti-cancer agents against breast cancer: synthesis, biological evaluation, molecular modelling studies. Material Science and Engineering C 99 (2019) 417-429. https://doi.org/10.1016/j.msec.2019.01.123. 10.1016/j.msec.2019.01.123 30889716

[79] Vyas K. Rathod M. Patel M.M.. Insight on nano drug delivery systems with targeted therapy in treatment of oral cancer. Nanomedicine: Nanotechnology, Biology, and Medicine 49 (2023) 102662. https://doi.org/10.1016/j.nano.2023.102662. 10.1016/j.nano.2023.102662 36746272

[80] Latorre A. Latorre A. Castellanos M. Lafuente-Gómez N. Diaz C.R. Crespo-Barreda A. Lecea M. Cordani M. Martín-Duque P. Somoza Á.. Albumin-based nanostructures for uveal melanoma treatment. Nanomedicine: Nanotechnology, Biology, and Medicine 35 (2021) 102391. https://doi.org/10.1016/j.nano.2021.102391. 10.1016/j.nano.2021.102391 33794371

[81] Caesar L.K. Cech N.B.. A Review of the Medicinal Uses and Pharmacology of Ashitaba. Planta Medica 82 (2016) 1236-1245. https://doi.org/10.1055/s-0042-110496. 10.1055/s-0042-110496 27399234

[82] Wu F. Zhu J. Li G. Wang J. Veeraraghavan V.P. Mohan S.K. Zhan Q.. Biologically synthesized green gold nanoparticles from Siberian ginseng induce growth-inhibitory effect on melanoma cells (B16). Artificial Cells, Nanomedicine, and Biotechnology 47 (2019) 3297-3305. https://doi.org/10.1080/21691401.2019.1647224. 10.1080/21691401.2019.1647224 31379212

[83] Zahraie N. Perota G. Dehdari Vais R. Sattarahmady N.. Simultaneous chemotherapy/sonodynamic therapy of the melanoma cancer cells using a gold-paclitaxel nanostructure. Photodiagnosis and Photodynamic Therapy 39 (2022) 102991. https://doi.org/10.1016/j.pdpdt.2022.102991. 10.1016/j.pdpdt.2022.102991 35779857

[84] Brochez L. Meireson A. Chevolet I. Sundahl N. Ost P. Kruse V.. Challenging PD-L1 expressing cytotoxic T cells as a predictor for response to immunotherapy in melanoma. Nature Communications 9 (2018) 2921. https://doi.org/10.1038/s41467-018-05047-1. 10.1038/s41467-018-05047-1 PMC6062523 30050132

[85] Banstola A. Poudel K. Emami F. Ku S.K. Jeong J.-H. Kim J.O. Yook S.. Localized therapy using anti-PD-L1 anchored and NIR-responsive hollow gold nanoshell (HGNS) loaded with doxorubicin (DOX) for the treatment of locally advanced melanoma. Nanomedicine: Nanotechnology, Biology, and Medicine 33 (2021) 102349. https://doi.org/10.1016/j.nano.2020.102349. 10.1016/j.nano.2020.102349 33359414

[86] Farahavar G. Abolmaali S.S. Nejatollahi F. Safaie A. Javanmardi S. Zadeh H.K. Yousefi R. Nadgaran H. Mohammadi-Samani S. Tamaddon A.Md. Ahadian S.. Single-chain antibody-decorated Au nanocages@liposomal layer nanoprobes for targeted SERS imaging and remote-controlled photothermal therapy of melanoma cancer cells. Materials Science & Engineering C 124 (2021) 112086. https://doi.org/10.1016/j.msec.2021.112086. 10.1016/j.msec.2021.112086 33947576

[87] Lopes J. Coelho J.M.P. Vieira P.M.C. Viana A.S. Gaspar M.M. Reis C.. Preliminary Assays towards Melanoma Cells Using Phototherapy with Gold-Based Nanomaterials. Nanomaterial 10 (2020) 1536. https://doi.org/10.3390/nano10081536. 10.3390/nano10081536 PMC7466595 32764377

[88] Shanei A. Akbari-Zadeh H. Attaran N. Salamat M.R. Baradaran-Ghahfarokhi M.. Effect of targeted gold nanoparticles size on acoustic cavitation: An in vitro study on melanoma cells. Ultrasonics 102 (2020) 106061-106071. https://doi.org/10.1016/j.ultras.2019.106061. 10.1016/j.ultras.2019.106061 31948804

[89] Perota G. Zahraie N. Dehdari Vais R. Zare M.H. Sattarahmady N.. Au/TiO2 nanocomposite as a triple-sensitizer for 808 and 650 nm phototherapy and sonotherapy: Synergistic therapy of melanoma cancer in vitro. Journal of Drug Delivery Science and Technology 76 (2022) 103787. https://doi.org/10.1016/j.jddst.2022.103787. 10.1016/j.jddst.2022.103787

[90] Soratijahromia E. Mohammadia S. Dehdari Vais R. Azarpira N. Sattarahmady N.. Photothermal/sonodynamic therapy of melanoma tumor by a gold/manganese dioxide nanocomposite: In vitro and in vivo studies. Photodiagnosis and Photodynamic Therapy 31 (2020) 101846. https://doi.org/10.1016/j.pdpdt.2020.101846. 10.1016/j.pdpdt.2020.101846 32492518

[91] Singh R.P. Reddy C.R.K.. Seaweed-microbial interactions: key functions of seaweed-associated bacteria. FEMS Microbiology Ecology 88 (2014) 213-230. https://doi.org/10.1111/1574-6941.12297. 10.1111/1574-6941.12297 24512602

[92] Acharya D. Satapathy S. Somu P. Parida U.K. Mishra G.. Apoptotic effect and anticancer activity of biosynthesized silver nanoparticles from marine algae Chaetomorpha linum extract against human colon cancer cell HCT-116. Biological Trace Element Research 199 (2021) 1812-1822. https://doi.org/10.1007/s12011-020-02304-7. 10.1007/s12011-020-02304-7 32743762

[93] Abdel-fattah W.I. Ali G.W.. On the anti-cancer activities of silver nanoparticles. Journal of Applied Biotechnology & Bioengineering 5 (2018) 3-7. https://doi.org/10.15406/jabb.2018.05.00116. 10.15406/jabb.2018.05.00116

[94] Sharma A. Goyal A.K. Rath G.. Recent advances in metal nanoparticles in cancer therapy. Journal of Drug Target 26 (2018) 617-632. https://doi.org/10.1080/1061186X.2017.1400553. 10.1080/1061186X.2017.1400553 29095640

[95] Shahriari M. Sedigh M.A. Mahdavian Y. Mahdigholizad S. Pirhayati M. Karmakar B. Veisi H.. In situ supported Pd NPs on biodegradable chitosan/agarose modified magnetic nanoparticles as an effective catalyst for the ultrasound assisted oxidation of alcohols and activities against human breast cancer. International Journal of Biological Macromolecules 172 (2021) 55-65. https://doi.org/10.1016/j.ijbiomac.2021.01.037. 10.1016/j.ijbiomac.2021.01.037 33444653

[96] Zangeneh M.M. Bovandi S. Gharehyakheh S. Zangeneh A. Irani P.. Green synthesis and chemical characterization of silver nanoparticles obtained using allium saralicum aqueous extract and survey of in vitro antioxidant, cytotoxic, antibacterial and antifungal properties. Applied Organometallic Chemistry 33 (2019) e4961. https://doi.org/10.1002/aoc.4961. 10.1002/aoc.4961

[97] Manikandan R. Anjali R. Beulaja M. Prabhu N.M. Koodalingam A. Saiprasad G. Chitra P. Arumugam M.. Synthesis, characterization, antiproliferative and wound healing activities of silver nanoparticles synthesized from Caulerpa scalpelliformis. Process Biochemistry 79 (2019) 135-141. https://doi.org/10.1016/j.procbio.2019.01.013. 10.1016/j.procbio.2019.01.013

[98] Zhao Z. Fang L. Lv D. Chen L. Zhang B. Wu D.. Design and synthesis of Ag NPs/chitosan-starch nano-biocomposite as a modern anti-human malignant melanoma drug. International Journal of Biological Macromolecules 236 (2023) 123823. https://doi.org/10.1016/j.ijbiomac.2023.123823. 10.1016/j.ijbiomac.2023.123823 36842739

[99] Patil P.M. Poddar N. Parihar N. Sen S. Mohapatra P. Murty U.S. Pemmaraju D.B.. Optoresponsive Pheophorbide-Silver based organometallic nanomaterials for high efficacy multimodal theranostics in Melanoma. Chemical Engineering Journal 470 (2023) 144110. https://doi.org/10.1016/j.cej.2023.144110. 10.1016/j.cej.2023.144110

[100] Bunyatova U. Ben Hammouda M. Zhang J.. Novel light-driven functional AgNPs induce cancer death at extra low concentrations. Scientific Report 11 (2021) 13258. https://doi.org/10.1038/s41598-021-92689-9. 10.1038/s41598-021-92689-9 PMC8225844 34168242

[101] Capanema N.S.V. Carvalho I.C. Mansur A.A.P. Carvalho S.M. Lage A.P. Mansur H.S.. Hybrid Hydrogel Composed of Carboxymethylcellulose–Silver Nanoparticles–Doxorubicin for Anticancer and Antibacterial Therapies against Melanoma Skin Cancer Cells. ACS Applied Nano Materials 2 (2019) 7393-7408. https://doi.org/10.1021/acsanm.9b01924. 10.1021/acsanm.9b01924

[102] Valenzuela-Salas L.M. Girón-Vázquez N.G. García-Ramos J.C. Torres-Bugarín O. Gómez C. Pestryakov A. Villarreal-Gómez L.J. Toledano-Magaña Y. Bogdanchikova N.. Antiproliferative and Antitumour Effect of Nongenotoxic Silver Nanoparticles on Melanoma Models. Oxidative Medicine and Cellular Longevity 2019 (2019) 4528241. https://doi.org/10.1155/2019/4528241. 10.1155/2019/4528241 31428226 PMC6683800

[103] Halevas E.G. Pantazaki A.A.. Copper nanoparticles as therapeutic anticancer agents. Nanomedicine and Nanotechnology Journal 2 (2018) 119. https://ikee.lib.auth.gr/record/315077/files/Halevas%20Pantazaki.

[104] Ma B. Wang S. Liu F. Zhang S. Duan J. Li Z. Kong Y. Sang Y. Liu H. Bu W. Li L.. Self-Assembled copper-amino acid nanoparticles for in situ glutathione “AND” H2O2 sequentially triggered chemodynamic therapy. Journal of the American Chemical Society 141 (2019) 849-857. https://doi.org/10.1021/jacs.8b08714. 10.1021/jacs.8b08714 30541274

[105] Wang Y. Wu W. Liu J. Manghnani P.N. Hu F. Ma D. Teh C. Wang B.o. Liu B.. Cancer-cell-activated photodynamic therapy assisted by Cu(II)-based metalorganic framework. ACS Nano 13 (2019) 6879-6890. https://doi.org/10.1021/acsnano.9b01665. 10.1021/acsnano.9b01665 31194910

[106] Lin L.-S. Huang T. Song J. Ou X.-Y. Wang Z. Deng H. Tian R. Liu Y. Wang J.-F. Liu Y. Yu G. Zhou Z. Wang S. Niu G. Yang H.-H. Chen X.. Synthesis of copper peroxide nanodots for H2O2 self-supplying chemodynamic therapy. Journal of the American Chemical Society 141 (2019) 9937-9945. https://doi.org/10.1021/jacs.9b03457. 10.1021/jacs.9b03457 31199131

[107] Song H. Xu Q. Zhu Y. Zhu S. Tang H. Wang Y. Ren H. Zhao P. Qi Z. Zhao S.. Serum adsorption, cellular internalization and consequent impact of cuprous oxide nanoparticles on uveal melanoma cells: implications for cancer therapy. Nanomedicine 10 (2015) 3547-3562. https://doi.org/10.2217/nnm.15.178. 10.2217/nnm.15.178 26467678

[108] Huo M. Wang L. Wang Y. Chen Y. Shi J., Nanocatalytic Tumor Therapy by Single-Atom Catalysts. ACS Nano 13 (2019) 2643-2653. https://doi.org/10.1021/acsnano.9b00457. 10.1021/acsnano.9b00457 30753056

[109] Yang B. Chen Y. Shi J., Nanocatalytic Medicine. Advanced Materials 31 (2019) e1901778. https://doi.org/10.1002/adma.201901778. 10.1002/adma.201901778 31328844

[110] Fang T. Ma S. Wei Y. Yang J. Zhang J. Shen Q.. Catalytic immunotherapy-photothermal therapy combination for melanoma by ferroptosis-activating vaccine based on artificial nanoenzyme. Materials Today Chemistry 27 (2023) 101308. https://doi.org/10.1016/j.mtchem.2022.101308. 10.1016/j.mtchem.2022.101308

[111] Liu Y. Xu C.-F. Iqbal S. Yang X.-Z. Wang J.. Responsive nanocarriers as an emerging platform for cascaded delivery of nucleic acids to cancer. Advanced Drug Delivery Reviews 115 (2017) 98-114. https://doi.org/10.1016/j.addr.2017.03.004. 10.1016/j.addr.2017.03.004 28396204

[112] Chen J. Zhu Y. Kaskel S.. Porphyrin-based metal-organic frameworks for biomedical applications. Angewandte Chemie International Edition 60 (2021) 5010-5035. https://doi.org/10.1002/anie.201909880. 10.1002/anie.201909880 31989749 PMC7984248

[113] Mukherjee S. Kotcherlakota R. Haque S. Haque S. Bhattacharya D. Kumar J.M. Chakravarty S. Patra C.R.. Improved delivery of doxorubicin using rationally designed PEGylated platinum nanoparticles for the treatment of melanoma. Material Sciences & Engineering C 108 (2020) 110375. https://doi.org/10.1016/j.msec.2019.110375. 10.1016/j.msec.2019.110375 31924026

[114] Xue W.L. Zhao X.N. Gao D.W. Gao F.M. Wang Z. Liu Y.P. Zhang X. Luoa L. Liua Z.. Octreotide acetate-templated self-assembly Pt nanoparticles and their anti-tumor efficacy. RSC Advances 5 (2015) 42186-42192. https://doi.org/10.1039/C5RA02921J. 10.1039/C5RA02921J

[115] Ling P.H. Lei J.P. Jia L. Ju H.X.. Platinum nanoparticles encapsulated metal-organic frameworks for the electrochemical detection of telomerase activity. Chemical Communications 52 (2016) 1226-1229. https://doi.org/10.1039/C5CC08418K. 10.1039/C5CC08418K 26612011

[116] Dasari S. Tchounwo P.B.. Cisplatin in cancer therapy: molecular mechanisms of action. European Journal of Pharmacology 740 (2014) 364-378. https://doi.org/10.1016/j.ejphar.2014.07.025. 10.1016/j.ejphar.2014.07.025 25058905 PMC4146684

[117] Johnstone T.C. Park G.Y. Lippard S.J.. Understanding and improving platinum anticancer drugs-phenanthriplatin. Anticancer Research 34 (2014) 471-476. https://pubmed.ncbi.nlm.nih.gov/24403503/. 24403503 PMC3937549

[118] Pedone D. Moglianetti M. Bardi G. Pompa P.P. De Luca E.. Platinum nanoparticles in nanobiomedicine. Chemical Society Reviews journal 46 (2017) 53-69. https://doi.org/10.1039/c7cs00152e. 10.1039/c7cs00152e 28696452

[119] Salehi F. Daneshvar F. Karimi M. Vais R.D. Mosleh-Shirazi M.A. Sattarahmady N.. Enhanced melanoma cell-killing by combined phototherapy/radiotherapy using a mesoporous platinum nanostructure. Photodiagnosis and Photodynamic Therapy 28 (2019) 300-307. https://doi.org/10.1016/j.pdpdt.2019.10.001. 10.1016/j.pdpdt.2019.10.001 31606514

[120] Slowing I.I. Trewyn B.G. Giri S. Lin V.S.Y.. Mesoporous silica nanoparticles for drug delivery and biosensing applications. Advanced Functional Materials 17 (2007) 1225-1236. https://doi.org/10.1002/adfm.200601191. 10.1002/adfm.200601191

[121] Truong N.P. Whittaker M.R. Mak C.W. Davis T.P.. The importance of nanoparticle shape in cancer drug delivery. Expert Opinion on Drug Delivery 12 (2015) 129-142. https://doi.org/10.1517/17425247.2014.950564. 10.1517/17425247.2014.950564 25138827

[122] Jeyaraj M. Gurunathan S. Qasim M. Kang M. Kim J.. A comprehensive review on the synthesis, characterization, and biomedical application of platinum nanoparticles. Nanomaterials 9 (2019) 2019. https://doi.org/10.3390/nano9121719. 10.3390/nano9121719 PMC6956027 31810256

[123] Yang S. Chen L. Zhou X. Sun P. Fu L. You Y. Xu M. You Z. Kai G. He C.. Tumor-targeted biodegradable multifunctional nanoparticles for cancer theranostics. Chemical Engineering Journal 378 (2019) 122171. https://doi.org/10.1016/j.cej.2019.122171. 10.1016/j.cej.2019.122171

[124] Pourmadadi M. Abbasi P. Eshaghi Md.M. Bakhshi A. Manicum A.-L.E. Rahdar A. Pandey S. Jadoun S. Díez-Pascual A.M.. Curcumin delivery and co-delivery based on nanomaterials as an effective approach for cancer therapy. Journal of Drug Delivery Science and Technology 78 (2022) 103982. https://doi.org/10.1016/j.jddst.2022.103982. 10.1016/j.jddst.2022.103982

[125] Ghazaeian M. Khorsandi K. Hosseinzadeh R. Naderi A. Abrahamse H.. Curcumin–silica nanocomplex preparation, hemoglobin and DNA interaction and photocytotoxicity against melanoma cancer cells. Journal of Biomolecular Structure and Dynamics 39 (2021) 6606-6616. https://doi.org/10.1080/07391102.2020.1802342. 10.1080/07391102.2020.1802342 32762410

[126] Idris N.M. Gnanasammandhan M.K. Zhang J. Ho P.C. Mahendran R. Zhang Y.. In vivo photodynamic therapy using upconversion nanoparticles as remote-controlled nanotransducers. Nature Medicine 18 (2012) 1580-1585. https://doi.org/10.1038/nm.2933. 10.1038/nm.2933 22983397

[127] Kim S.E. Zhang L. Ma K. Riegman M. Chen F. Ingold I. Conrad M. Turker M.Z. Gao M. Jiang X. Monette S. Pauliah M. Gonen M. Zanzonico P. Quinn T. Wiesner U. Bradbury M.S. Overholtzer M.. Ultrasmall nanoparticles induce ferroptosis in nutrient-deprived cancer cells and suppress tumour growth. Nature Nanotechnology 11 (2016) 977-985. https://doi.org/0.1038/nnano.2016.164. 10.1038/nnano.2016.164 PMC5108575 27668796

[128] Wang H. Zhao R. Li Y. Liu H. Li F. Zhao Y. Nie G.. Aspect ratios of gold nanoshell capsules mediated melanoma ablation by synergistic photothermal therapy and chemotherapy. Nanomedicine: Nanotechnology, Biology and Medicine 12 (2016) 439-448. https://doi.org/10.1016/j.nano.2015.11.013. 10.1016/j.nano.2015.11.013 26711964

[129] Zhang Z. Zhang X. Zhao S. Feng W. Huang H. Ding L. Chen Y. Chen B.. Engineering 2D silicene-based core/shell nanomedicine for GSDME-induced synergistic pyroptosis and photonic hyperthermia of melanoma carcinoma. Chemical Engineering Journal 454 (2023) 140175. https://doi.org/10.1016/j.cej.2022.140175. 10.1016/j.cej.2022.140175

[130] Sapino S. Ugazio E. Gastaldi L. Miletto I. Berlier G. Zonari D. Oliaro-Bosso S.. Mesoporous silica as topical nanocarriers for quercetin: characterization and in vitro studies. European Journal of Pharmaceutics and Biopharmaceutics 89 (2015) 116-125. https://doi.org/10.1016/j.ejpb.2014.11.022. 10.1016/j.ejpb.2014.11.022 25478737

[131] Gurr R.J.. Ultrafine titanium dioxide particles in the absence of photoactivation can induce oxidative damage to human bronchial epithelial cells. Toxicology 213 (2005) 66-73. https://doi.org/10.1016/j.tox.2005.05.007. 10.1016/j.tox.2005.05.007 15970370

[132] Jafari S. Mahyad B. Hashemzadeh H. Janfaza S. Gholikhani T. Tayebi L.. Biomedical applications of TiO2 nanostructures: recent advances. International Journal of Nanomedicine 15 (2020) 3447-3470. https://doi.org/10.2147/IJN.S249441. 10.2147/IJN.S249441 32523343 PMC7234979

[133] Meng A. Zhang L. Cheng B. Yu J.. Dual Cocatalysts in TiO2 Photocatalysis. Advanced Materials 31 (2019) 1807660. https://doi.org/10.1002/adma.201807660. 10.1002/adma.201807660 31148244

[134] Zarzzeka C. Goldoni J. Marafon F. Sganzerla W.G. Forster-Carneiro T. Bagatini M.D. Maria L. Colpini S.. Use of titanium dioxide nanoparticles for cancer treatment: A comprehensive review and bibliometric analysis. Biocatalysis and Agricultural Biotechnology 50 (2023) 102710. https://doi.org/10.1016/j.bcab.2023.102710. 10.1016/j.bcab.2023.102710

[135] Deshpande S.S. Veeragoni D.K. Kongari L. Mamilla J. Misra S.. Synthesis of biocompatible chitosan coated TiO2-curcumin nanocomposites shows potent anticancer activity towards melanoma cancer cells. Journal of Drug Delivery Science and Technology 85 (2023) 104592. https://doi.org/10.1016/j.jddst.2023.104592. 10.1016/j.jddst.2023.104592

[136] Widder K.J. Marino P.A. Morris R.M. Howard D.P. Poore G.A. Senyei A.E.. Selective targeting of magnetic albumin microspheres to the Yoshida sarcoma: Ultrastructural evaluation of microsphere disposition. European Journal of Cancer and Clinical Oncology 19 (1983) 141-147. https://doi.org/10.1016/0277-5379(83)90409-1. 10.1016/0277-5379(83)90409-1 6682772

[137] Santhosh P.B. Ulrih N.P.. Multifunctional superparamagnetic iron oxide nanoparticles: promising tools in cancer theranostics. Cancer Letters 336 (2013) 8-17. https://doi:10.1016/j.canlet.2013.04.032. 10.1016/j.canlet.2013.04.032 23664890

[138] Plank C.. Nanomedicine: silence the target. Nature Nanotechnology 4 (2009) 544-545. https://doi.org/10.1038/nnano.2009.251. 10.1038/nnano.2009.251 19734920

[139] Rajan M. Krishnan P. Pradeepkumar P. Jeyanthinath M. Jeyaraj M. Ling M.P. Arulselvan P. Higuchi A. Munusamy M.A. Arumugam R. Benelli G. Muruganmo K. Kumar S.S.. Magneto-chemotherapy for cervical cancer treatment with camptothecin loaded Fe3O4 functionalized β-cyclodextrin nanovehicle. RSC Advances 7 (2017) 46271-46285, https://doi.org/10.1039/c7ra06615e. 10.1039/c7ra06615e

[140] Zheng W. Zhou Q. Yuan C.. Nanoparticles for Oral cancer diagnosis and therapy. Bioinorganic Chemistry and Applications 2021 (2021) 1-14. https://doi.org/10.1155/2021/9977131. 10.1155/2021/9977131 PMC8088384 33981334

[141] Geng H. Dai Q. Sun H. Zhuang L. Song A. Caruso F. Hao J. Cui J.. Injectable and sprayable polyphenol-based hydrogels for controlling hemostasis. ACS Applied Bio Materials 3 (2020) 1258-1266. https://doi.org/10.1021/acsabm.9b01138. 10.1021/acsabm.9b01138 35019326

[142] Zhang B. Yao R. Hu C. Maitz M.F. Wu H. Liu K. Yang L. Luo R. Wang Y.. Epigallocatechin gallate mediated sandwich-like coating for mimicking endothelium with sustained therapeutic nitric oxide generation and heparin release. Biomaterials 269 (2020) 120418. https://doi:10.1016/j.biomaterials.2020.120418. 10.1016/j.biomaterials.2020.120418 33143876

[143] Guo J. Ping Y. Ejima H. Alt K. Meissner M. Richardson J.R. Yan Y. Peter K. von Elverfeldt D. Hagemeyer C.E. Caruso F.. Engineering multifunctional capsules through the assembly of metalphenolic networks”. Angewandte Chemie International Edition 53 (2014) 5546-5551. https://doi.org/10.1002/anie.201311136. 10.1002/anie.201311136 24700671

[144] Mu M. Liang X. Chuan D. Zhao S. Yu W. Fan R. Tong A. Zhao N. Han B. Guo G.. Chitosan coated pH-responsive metal-polyphenol delivery platform for melanoma chemotherapy. Carbohydrate Polymers 264 (2021) 118000. https://doi.org/10.1016/j.carbpol.2021.118000. 10.1016/j.carbpol.2021.118000 33910734

[145] Chuan D. Hou H. Wang Y. Mu M. Li J. Ren Y. Zhao N. Han B. Chen H. Guo G.. Multifunctional metal-polyphenol nanocomposite for melanoma targeted photo/chemodynamic synergistic therapy. Journal of Materials Science & Technology 152 (2023) 159-168. https://doi.org/10.1016/j.jmst.2022.12.039. 10.1016/j.jmst.2022.12.039

[146] Li P. Li Y. Fu R. Duan Z. Zhu C. Fan D.. NIR- and pH-responsive injectable nanocomposite alginate-graft-dopamine hydrogel for melanoma suppression and wound repair. Carbohydrate Polymers 314 (2023) 120899. https://doi.org/10.1016/j.carbpol.2023.120899. 10.1016/j.carbpol.2023.120899 37173039

[147] Iijima S.. Helical microtubules of graphitic carbon. Nature 354 (1991) 56-58. https://doi.org/10.1038/354056a0. 10.1038/354056a0

[148] Liu Z. Tabakman S. Welsher K. Dai H.. Carbon nanotubes in biology and medicine: in vitro and in vivo detection, imaging and drug delivery. Nano Research 2 (2009) 85-120. https://doi.org/10.1007/s12274-009-9009-8. 10.1007/s12274-009-9009-8 20174481 PMC2824900

[149] Peretz S. Regev O.. Carbon nanotubes as nanocarriers in medicine. Current Opinion in Colloid & Interface Science 17 (2012) 360-368. https://doi.org/10.1016/j.cocis.2012.09.001. 10.1016/j.cocis.2012.09.001

[150] Smart S.K. Cassady A.I. Lu G.Q. Martin D.J.. The biocompatibility of carbon nanotubes. Carbon 44 (2006) 1034-1047. https://doi.org/10.1016/j.carbon.2005.10.011. 10.1016/j.carbon.2005.10.011

[151] Parhi R.. Development and in Vitro evaluation of etoricoxib loaded transdermal film containing MWCNT. Journal of Advanced Applied Scientific Research 4 (2022) 1-8. https://doi.org/10.46947/joaasr442022439. 10.46947/joaasr442022439

[152] Cirillo G. Hampel S. Spizzirri U.G. Parisi O.I. Picci N. Iemma F.. Carbon nanotubes hybrid hydrogels in drug delivery: A perspective review. BioMed Research International 2014 (2014) 825017. https://doi.org/10.1155/2014/825017. 10.1155/2014/825017 24587993 PMC3918724

[153] Saleem J. Wang L. Chen C.. Carbon-based nanomaterials for cancer therapy via targeting tumor microenvironment. Advanced Healthcare Materials 20 (2018) 800525. https://doi.org/10.1002/adhm.201800525. 10.1002/adhm.201800525 30073803

[154] Chaudhuri P. Soni S. Sengupta S.. Single-walled carbon nanotube-conjugated chemotherapy exhibits increased therapeutic index in melanoma. Nanotechnology 21 (2010) 025100-025102. https://doi.org/10.1088/0957-4484/21/2/025102. 10.1088/0957-4484/21/2/025102 19955607

[155] Behzadpour N. Ranjbar A. Azarpira N. Sattarahmady N.. Development of a Composite of Polypyrrole-Coated Carbon Nanotubes as a Sonosensitizer for Treatment of Melanoma Cancer Under Multi-Step Ultrasound Irradiation. Ultrasound in Medicine & Biology 46 (2020) 2322-2334. https://doi.org/10.1016/j.ultrasmedbio.2020.05.003. 10.1016/j.ultrasmedbio.2020.05.003 32522457

[156] Yao J. Sun Y. Yang M. Duan Y.. Chemistry, physics and biology of graphene based nanomaterials: new horizons for sensing, imaging and medicine. Journal of Materials Chemistry 22 (2012) 14313. https://doi.org/10.1039/C2JM31632C. 10.1039/C2JM31632C

[157] Liua J. Donga J. Zhangb T. Peng Q.. Graphene-based nanomaterials and their potentials in advanced drug delivery and cancer therapy. Journal of Controlled Release 286 (2018) 64-73. https://doi.org/10.1016/j.jconrel.2018.07.034. 10.1016/j.jconrel.2018.07.034 30031155

[158] Lin J. Chen X. Huang P.. Graphene-based nanomaterials for bioimaging. Advanced Drug Delivery Reviews 105 (2016) 242-254. https://doi.org/10.1016/j.addr.2016.05.013. 10.1016/j.addr.2016.05.013 27233213 PMC5039069

[159] Hummers W.S. Offeman R.E.. Preparation of graphitic oxide. Journal of the American Chemical Society 80 (1958) 1339. https://doi.org/10.1021/ja01539a017. 10.1021/ja01539a017

[160] You S. Yu J. Sundqvist B. Talyzin A.V.. Solvation of graphite oxide in water–methanol binary polar solvents. Physica Status Solidi B 249 (2012) 2568-2571. https://doi.org/10.1002/pssb.201200059. 10.1002/pssb.201200059

[161] Hermanová S. Zarevúcká M. Bouša D. Pumera M. Sofer Z., Graphene oxide immobilized enzymes show high thermal and solvent stability. Nanoscale 7 (2015) 5852-5858. https://doi.org/10.1039/C5NR00438A. 10.1039/C5NR00438A 25757536

[162] McCallion C. Burthem J. Rees-Unwin K. Golovanov A. Pluen A.. Graphene in therapeutics delivery: problems, solutions and future opportunities. European Journal of Pharmaceutics and Biopharmaceutics 104 (2016) 235-250. https://doi.org/10.1016/j.ejpb.2016.04.015. 10.1016/j.ejpb.2016.04.015 27113141

[163] Liu Y. Zhong H. Qin Y. Zhang Y. Liu X. Zhang T.. Non-covalent hydrophilization of reduced graphene oxide used as a paclitaxel vehicle. RSC Advances 6 (2016) 30184-30193. https://doi.org/10.1039/C6RA04349F. 10.1039/C6RA04349F

[164] Chang H. Wu H.. Graphene-based nanomaterials: synthesis, properties, and optical and optoelectronic applications. Advanced Functional Materials 23 (2013) 1984-1997. https://doi.org/10.1002/adfm.201202460. 10.1002/adfm.201202460

[165] Stobinski L. Lesiak B. Malolepszy A. Mazurkiewicz M. Mierzwa B. Zemek J. Jiricek P. Bieloshapka I.. Graphene oxide and reduced graphene oxide studied by the XRD, TEM and electron spectroscopy methods. Journal of Electron Spectroscopy and Related Phenomena 195 (2014) 145-154. https://doi.org/10.1016/j.elspec.2014.07.003. 10.1016/j.elspec.2014.07.003

[166] Mokhtar M. Enein S.A.E. Hassaan M.Y. Morsy M.S. Khalil M.H.. Thermally reduced graphene oxide: synthesis, structural and electrical properties. International Journal of Nanoparticles and Nanotechnology 3 (2017) 008. https://doi.org/10.35840/2631-5084/5508. 10.35840/2631-5084/5508

[167] Nazir S. Khan Md.U.A. Al-Arjan W.S. Razak S.I.A. Javed A. Kadir Md.R.A.. Nanocomposite hydrogels for melanoma skin cancer care and treatment: In-vitro drug delivery, drug release kinetics and anti-cancer activities. Arabian Journal of Chemistry 14 (2021) 103120. https://doi.org/10.1016/j.arabjc.2021.103120. 10.1016/j.arabjc.2021.103120

[168] Zhan X. Teng W. Sun K. He J. Yang J. Tian J. Huang X. Zhou L. Zhou C.. CD47-mediated DTIC-loaded chitosan oligosaccharide-grafted nGO for synergistic chemo-photothermal therapy against malignant melanoma. Materials Science & Engineering C 123 (2021) 112014. https://doi.org/10.1016/j.msec.2021.112014. 10.1016/j.msec.2021.112014 33812633

[169] Bamburowicz-Klimkowska M. Malecki M. Bystrzejewski M. Kasprzak A. Grudzinski I.P.. Graphene-encapsulated iron nanoparticles as a non-viral vector for gene delivery into melanoma cells. Biochemical and Biophysical Research Communications 652 (2023) 84-87. https://doi.org/10.1016/j.bbrc.2023.02.042. 10.1016/j.bbrc.2023.02.042 36841098

[170] Guo Q. Li L. Guo G. Liu R. Einaga Y. Zhi J. Nanodiamonds Inhibit Cancer Cell Migration by Strengthening Cell Adhesion: Implications for Cancer Treatment. ACS Applied Materials & Interfaces 13 (2021) 9620-9629. https://doi.org/10.1021/acsami.0c21332. 10.1021/acsami.0c21332 33595291

[171] Nicolas J. Mura S. Brambilla D. Mackiewicz N. Couvreur P.. Design, functionalization strategies and biomedical applications of targeted biodegradable/biocompatible polymer-based nanocarriers for drug delivery. Chemical Society Reviews 42 (2013) 1147-1235. https://doi.org/10.1039/c2cs35265f. 10.1039/c2cs35265f 23238558

[172] Deirram N. Zhang C. Kermaniyan S.S. Johnston A.P.R. Such G.K.. pH-responsive polymer nanoparticles for drug delivery. Macromolecular Rapid Communications 40 (2019) 1800917. https://doi.org/10.1002/marc.201800917. 10.1002/marc.201800917 30835923

[173] Lee Y. Thompson D.. Stimuli-responsive liposomes for drug delivery. Wiley Interdisciplinary Reviews: Nanomedicine and Nanobiotechnology 9 (2017) e1450. https://doi.org/10.1002/wnan.1450. 10.1002/wnan.1450 PMC5557698 28198148

[174] Hu C.-M.J. Fang R.H. Luk B.T. Zhang L.. Polymeric nanotherapeutics: clinical development and advances in stealth functionalization strategies. Nanoscale 6 (2014) 65-75. https://doi.org/10.1039/C3NR05444F. 10.1039/C3NR05444F 24280870

[175] Kaur S. Prasad C. Balakrishnan B. Banerjee R.. Trigger responsive polymeric nanocarriers for cancer therapy. Biomaterial Science 3 (2015) 955-987. https://doi.org/10.1039/c5bm00002e. 10.1039/c5bm00002e 26221933

[176] Patel V.K. Srivastava R. Sharma A. Srivastava A.K. Singh S. Srivastava A.K. Kashyap P.L. Chakdar H. Pandiyan K. Kalra A. Saxena A.K.. Halotolerant Exiguobacterium profundum PHM11 tolerate salinity by accumulating L-proline and finetuning gene expression profiles of related metabolic pathways. Frontiers in Microbiology 9 (2018) 1-14. https://doi.org/10.3389/fmicb.2018.00423. 10.3389/fmicb.2018.00423 29662469 PMC5890156

[177] Abd Ellah N.H. Abouelmagd S.A.. Surface functionalization of polymeric nanoparticles for tumor drug delivery: approaches and challenges. Expert Opinion on Drug Delivery 14 (2017) 201-214. https://doi.org/10.1080/17425247.2016.1213238. 10.1080/17425247.2016.1213238 27426638

[178] Danhier F. Ansorena E. Silva J.M. Coco R. Le Breton A. Préat V.. PLGA-based nanoparticles: an overview of biomedical applications. Journal of Controlled Release 161 (2012) 505-522. https://doi.org/10.1016/j.jconrel.2012.01.043. 10.1016/j.jconrel.2012.01.043 22353619

[179] Cagel M. Grotz E. Bernabeu E. Moretton M.A. Chiappetta D.A., Doxorubicin: nanotechnological overviews from bench to bedside. Drug Discovery Today 22 (2017) 270-281. https://doi.org/10.1016/j.drudis.2016.11.005. 10.1016/j.drudis.2016.11.005 27890669

[180] Zielinska A. Carreir F. Oliveira A.M. Neves A. Pires B. Venkatesh D.N. Durazzo A. Lucarini M. Eder P. Silva A.M. Santini A. Souto E.B.. Polymeric nanoparticles: production, characterization, toxicology and ecotoxicology. Molecules 25 (2020). https://doi.org/10.3390/molecules25163731. 10.3390/molecules25163731 PMC7464532 32824172

[181] Mehandole A. Walke N. Mahajan S. Aalhate M. Maji I. Gupta U. Mehra N.K. Singh P.K.. Core-shell type lipidic and polymeric nanocapsules: the transformative multifaceted delivery systems. AAPS PharmSciTech 24 (2023) 50. https://doi.org/10.1208/s12249-023-02504-z. 10.1208/s12249-023-02504-z 36703085

[182] Pohlmann A.R. Detoni C.B. Paese K. Coradini K. Beck R.C. Guterres S.S., Polymeric nanocapsules for topical delivery, in Dragicevic N. Maibach H. (Eds.)., Percutaneous Penetration Enhancers Chemical Methods in Penetration Enhancement: Nanocarriers, Springer, Berlin, Heidelberg. 2016, p. 201-221. https://doi.org/10.1007/978-3-662-47862-2_13. 10.1007/978-3-662-47862-2_13

[183] Pan R. Zeng Y. Liu G. Wei Y. Xu Y. Tao L., Curcumin-polymer conjugates with dynamic boronic acid ester linkages for selective killing of cancer cells. Polymer Chemistry 11 (2020) 1321-1326. https://doi.org/10.1039/C9PY01596E. 10.1039/C9PY01596E

[184] Scopel R. Falcão M.A. Cappellari A.R. Morrone F.B. Guterres S.S. Cassel E. Kasko A.M. Vargas R.M.F.. Lipid-polymer hybrid nanoparticles as a targeted drug delivery system for melanoma treatment. International Journal of Polymeric Materials and Polymeric Biomaterials 71 (2022) 127-138. https://doi.org/10.1080/00914037.2020.1809406. 10.1080/00914037.2020.1809406

[185] Song M. Zhu B. Qiu S. Tian J. Li J. Chen D. Liu C.. Codelivery of afuresertib and celecoxib by IL4RPep-1-targeting nanoparticles for effective treatment against melanoma. Applied Materials Today 33 (2023) 101868. https://doi.org/10.1016/j.apmt.2023.101868. 10.1016/j.apmt.2023.101868

[186] Mishra B. Patel Bhavesh B. Tiwari Sanjay. Colloidal nanocarriers: a review on formulation technology, types and applications toward targeted drug delivery. Nanomedicine: Nanotechnology Biology and Medicine 6 (2010) 9-24. https://doi.org/10.1016/j.nano.2009.04.008. 10.1016/j.nano.2009.04.008 19447208

[187] Mudshinge S.R. Deore A.B. Patil S. Bhalgat C.M.. Nanoparticles: Emerging carriers for drug delivery. Saudi Pharmaceutical Journal 19 (2011) 129-141. https://doi.org/10.1016/j.jsps.2011.04.001. 10.1016/j.jsps.2011.04.001 23960751 PMC3744999

[188] Hsu H.J. Bugno J. Lee S.-R. Hong S.. Dendrimer-based nanocarriers: a versatile platform for drug delivery. Wiley Interdisciplinary Reviews-Nanomedicine and Nanobiotechnology 9 (2017). https://doi.org/10.1002/wnan.1409. 10.1002/wnan.1409 27126551

[189] Jiang G. Li R. Tang J. Ma Y. Hou X. Yang C. Guo W. Xin Y. Liu Y.. Formulation of temozolomide-loaded nanoparticles and their targeting potential to melanoma cells. Oncology Reports 37 (2017) 995-1001. https://doi.org/10.3892/or.2016.5342. 10.3892/or.2016.5342 28035395

[190] Cheng C.-C. Chang F.-C. Kao W.-Y. Hwang S.-M. Liao L.-C. Chang Y.-J. Liang M.-C. Chen J.-K. Lee D.-J.. Highly efficient drug delivery systems based on functional supramolecular polymers: In vitro evaluation. Acta Biomaterialia 33 (2016) 194-202. https://doi.org/10.1016/j.actbio.2016.01.018. 10.1016/j.actbio.2016.01.018 26796210

[191] Bhat S. Guddadar V.. Targeted therapy in chronic diseases treatment by nanomaterial based drug delivery. International Journal of Advanced Research in Science, Communication and Technology 2 (2021) 141-145, https://doi.org/10.48175/ijarsct-799. 10.48175/ijarsct-799

[192] Tokarska K. Lamch Ł. Piechota B. Żukowski K. Chudy M. Wilk K.A. Brzózka Z.. Co-delivery of IR-768 and daunorubicin using mPEG-b-PLGA micelles for synergistic enhancement of combination therapy of melanoma. Journal of Photochemistry & Photobiology, B: Biology 211 (2020) 111981. https://doi.org/10.1016/j.jphotobiol.2020.111981. 10.1016/j.jphotobiol.2020.111981 32862088

[193] Wu H.-Q. Wang C.. Biodegradable Smart Nanogels: A New Platform for Targeting Drug Delivery and Biomedical Diagnostics. Langmuir 32 (2016) 6211-6225. https://doi.org/10.1021/acs.langmuir.6b00842. 10.1021/acs.langmuir.6b00842 27255455

[194] Chen W. Hou Y. Tu Z. Gao L. Haag R.. pH-degradable PVA-based nanogels via photo-crosslinking of thermo-preinduced nanoaggregates for controlled drug delivery. Journal of Controlled Release 16 (2016) 31116. https://doi.org/10.1016/j.jconrel.2016.10.032. 10.1016/j.jconrel.2016.10.032 27810557

[195] Tran P.H. Duan W. Lee B.-J. Tran T.T.. Nanogels for skin cancer therapy via transdermal delivery: current designs. Current Drug Metabolism 20 (2019) 575-582, https://doi.org/10.2174/1389200220666190618100030. 10.2174/1389200220666190618100030 31237201

[196] Kousalov´a J. Etrych T.. Polymeric nanogels as drug delivery systems. Physiological Research 67 (2018). https://doi.org/10.33549/physiolres.933979. 10.33549/physiolres.933979 30379552

[197] Zhao W. Hu J. Gao W.. Glucose Oxidase-Polymer Nanogels for Synergistic Cancer-Starving and Oxidation Therapy. ACS Applied Materials & Interfaces 9 (2017) 23528-23535. https://doi.org/10.1021/acsami.7b06814. 10.1021/acsami.7b06814 28650613

[198] Veisi H. Varshosaz J. Rostami M. Mirian M.. Thermosensitive TMPO-oxidized lignocellulose/cationic agarose hydrogel loaded with deferasirox nanoparticles for photothermal therapy in melanoma. International Journal of Biological Macromolecules 238 (2023) 124126. https://doi.org/10.1016/j.ijbiomac.2023.124126. 10.1016/j.ijbiomac.2023.124126 36944379

[199] García-Pinel B. Porras-Alcalá C. Ortega-Rodríguez A. Sarabia F. Prados J. Melguizo C. López-Romero J.M.. Lipid-based nanoparticles: application and recent advances in cancer treatment. Nanomaterials 9 (2019) 638. https://doi.org/0.3390/nano9040638. 10.3390/nano9040638 31010180 PMC6523119

[200] Kumar R.. Lipid-based nanoparticles for drug-delivery systems, in Mohapatra S.S. Ranjan S. Dasgupta N. Mishra R.K. Thomas S. (Eds.)., Nanocarriers for Drug Delivery, Elsevier, 2019, p. 249-284. https://doi.org/10.1016/B978-0-12-814031-4.00003-9. 10.1016/B978-0-12-814031-4.00003-9

[201] Sah A.K. Vyas A. Suresh P.K. Gidwani B.. Application of nanocarrier based drug delivery system in treatment of oral cancer. Artificial Cells, Nanomedicine, and Biotechnology 46 (2018) 650-657. https://doi.org/10.1080/21691401.2017.1373284. 10.1080/21691401.2017.1373284 28880679

[202] Pattni B.S. Chupin V.V. Torchilin V.P.. New Developments in Liposomal Drug Delivery. Chemical Reviews 115 (2015) 10938-10966. https://doi.org/10.1021/acs.chemrev.5b00046. 10.1021/acs.chemrev.5b00046 26010257

[203] Akbarzadeh A. Rezaei-Sadabady R. Davaran S. Joo S.W. Zarghami N. Hanifehpour Y. Samiei Md. Kouhi Md. Nejati-Koshki K.. Liposome: classification, preparation, and applications. Nanoscale Research Letters 8 (2013) 102. https://doi.org/10.1186/1556-276X-8-102. 10.1186/1556-276X-8-102 23432972 PMC3599573

[204] Krishnan V. Mitragotri S.. Nanoparticles for topical drug delivery: potential for skin cancer treatment. Advanced Drug Delivery Reviews 153 (2020) 87-108. https://doi.org/10.1016/j.addr.2020.05.011. 10.1016/j.addr.2020.05.011 32497707

[205] Daraee H. Etemadi A. Kouhi M. Alimirzalu S. Akbarzadeh A.. Application of liposomes in medicine and drug delivery. Artificial Cells, Nanomedicine, and Biotechnology 44 (2016) 381-391. https://doi.org/10.3109/21691401.2014.953633. 10.3109/21691401.2014.953633 25222036

[206] Ahmadi A. Hosseini-Nami S. Abed Z. Beik J. Aranda-Lara L. Samadian H. Morales-Avila E. Jaymand M. Shakeri-Zadeh A.. Recent advances in ultrasound triggered drug delivery through lipid-based nanomaterials. Drug Discovery Today 25 (2020) 2182-2200. https://doi.org/10.1016/j.drudis.2020.09.026. 10.1016/j.drudis.2020.09.026 33010479

[207] Jahani V. Yazdani M. Badiee A. Jaafari M.R. Arabi L.. Liposomal celecoxib combined with dendritic cell therapy enhances antitumor efficacy in melanoma. Journal of Controlled Release 354 (2023) 453-464. https://doi.org/10.1016/j.jconrel.2023.01.034. 10.1016/j.jconrel.2023.01.034 36649743

[208] Lee E.H. Lim S.J. Lee M.K.. Chitosan-coated liposomes to stabilize and enhance transdermal delivery of indocyanine green for photodynamic therapy of melanoma. Carbohydrate Polymer 224 (2019) 115-143. https://doi.org/10.1016/j.carbpol.2019.115143. 10.1016/j.carbpol.2019.115143 31472877

[209] Mishra H. Mishra P.K. Iqbal Z. Jaggi M. Madaan A. Bhuyan K. Gupta N. Gupta N. Vats K. Verma R. Talegaonkar S.. Co-Delivery of Eugenol and Dacarbazine by Hyaluronic Acid-Coated Liposomes for Targeted Inhibition of Survivin in Treatment of Resistant Metastatic Melanoma. Pharmaceutics 11 (2019) 163. https://doi.org/10.3390/pharmaceutics11040163. 10.3390/pharmaceutics11040163 30987266 PMC6523131

[210] Cadinoiu A.N. Rata D.M. Atanase L.I. Daraba O.M. Gherghel D. Vochita G. Popa M.. Aptamer-Functionalized Liposomes as a Potential Treatment for Basal Cell Carcinoma. Polymers 11 (2019) 1515. https://doi.org/10.3390/polym11091515. 10.3390/polym11091515 31540426 PMC6780726

[211] Hadjidemetriou M. Al-Ahmady Z. Buggio M. Swift J. Kostarelos K.. A novel scavenging tool for cancer biomarker discovery based on the blood-circulating nanoparticle protein corona. Biomaterials 188 (2019) 118-129. https://doi.org/10.1016/j.biomaterials.2018.10.011. 10.1016/j.biomaterials.2018.10.011 30343255

[212] Yeo P.L. Lim C.L. Chye S.M. Ling A.P.K. Koh R.Y.. Niosomes: a review of their structure, properties, methods of preparation, and medical applications. Asian Biomedicine 11 (2017) 301-314. https://doi.org/10.1515/abm-2018-0002. 10.1515/abm-2018-0002

[213] Gharbavi M. Johari B. Mousazadeh N. Rahimi B. Leilan M.P. Eslami S.S. Sharafi A.. Hybrid of niosomes and bio-synthesized selenium nanoparticles as a novel approach in drug delivery for cancer treatment. Molecular Biology Reports 47 (2020) 6517-6529, https://doi.org/10.1007/s11033-020-05704-z. 10.1007/s11033-020-05704-z 32767222

[214] Chermahini S.H. Najafi R.B.. Niosome encapsulated fluorouracil as drug delivery system to basal-cell skin Cancer. Journal of Nanoscience and Nanomedicine 3 (2019) 1-4. https://www.pulsus.com/scholarly-articles/niosome-encapsulated-fluorouracil-as-drug-delivery-system-to-basalcell-skin-cancer-5149.html.

[215] Cristiano M.C. Froiio F. Spaccapelo R. Mancuso A. Nisticò S.P. Udongo B.P. Fresta M. Paolino D.. Sulforaphane-Loaded Ultradeformable Vesicles as A Potential Natural Nanomedicine for the Treatment of Skin Cancer Diseases. Pharmaceutics 12 (2019) 6. https://doi.org/10.3390/pharmaceutics12010006. 10.3390/pharmaceutics12010006 31861672 PMC7023209

[216] Mukherjee P.K. Harwansh R.K. Bhattacharyya S.. Bioavailability of herbal products: approach toward improved pharmacokinetics, in Mukherjee P. K. (Ed.)., Evidence-Based Validation of Herbal Medicine, Elsevier, Boston, 2015, p. 217-245. https://doi.org/10.1016/B978-0-12-800874-4.00010-6. 10.1016/B978-0-12-800874-4.00010-6

[217] Yang L. Wang Zheran Wang Ju Jiang Weihua Jiang Xuewei Bai Zhaoshi He Yunpeng Jiang Jianqi Wang Dongkai Yang Li. Doxorubicin conjugated functionalizable carbon dots for nucleus targeted delivery and enhanced therapeutic efficacy. Nanoscale 8 (2016) 6801-6809. https://doi.org/10.1039/C6NR00247A. 10.1039/C6NR00247A 26957191

[218] Xu Z. Zhu Shaojia Wang Mingwei Li Yongjun Shi Ping Huang Xiaoyu. Delivery of paclitaxel using PEGylated graphene oxide as a nanocarrier. ACS Applied Materials & Interfaces 7 (2015) 1355-1363. https://doi.org/10.1021/am507798d. 10.1021/am507798d 25546399

[219] Mehnert W. M¨ader K.. Solid lipid nanoparticles: production, characterization and applications. Advanced Drug Delivery Reviews 64 (2012) 83-101, https://doi.org/10.1016/j.addr.2012.09.021. 10.1016/j.addr.2012.09.021 11311991

[220] Abak A. Abhari A. Rahimzadeh S.. Exosomes in cancer: small vesicular transporters for cancer progression and metastasis, biomarkers in cancer therapeutics. Peer Reviewed Journal 6 (2018) e4763. https://doi.org/10.7717/peerj.4763. 10.7717/peerj.4763 PMC5983002 29868251

[221] Jabbari N. Karimipour M. Khaksar M. Akbariazar E. Heidarzadeh M. Mojarad B. Aftab H. Rahbarghazi R. Rezaie J.. Tumor-derived extracellular vesicles: insights into bystander effects of exosomes after irradiation. Lasers in Medical Science 35 (2020) 531-545. https://doi.org/10.1007/s10103-019-02880-8. 10.1007/s10103-019-02880-8 31529349

[222] Thery C. Ostrowski M. Segura E.. Membrane vesicles as conveyors of immune responses. Nature Reviews Immunology 9 (2009) 581-593. https://doi.org/10.1038/nri2567. 10.1038/nri2567 19498381

[223] Huyan T. Li H. Peng H. Chen J. Yang R. Zhang W. Li Q.. Extracellular vesicles – advanced nanocarriers in cancer therapy: progress and achievements. International Journal Nanomedicine 15 (2020) 6485-502. https://doi.org/10.2147/IJN.S238099. 10.2147/IJN.S238099 PMC7457829 32922012

[224] Millard M. Yakavets I. Piffoux M. Brun A. Gazeau F. Guigner J.-M. Jasniewski J. Lassalle H.-P. Wilhelm C. Bezdetnaya L.. mTHPC-loaded extracellular vesicles outperform liposomal and free mTHPC formulations by an increased stability, drug delivery efficiency and cytotoxic effect in tridimensional model of tumors. Drug Delivery 25 (2018) 1790-1801. https://doi.org/10.1080/10717544.2018.1513609. 10.1080/10717544.2018.1513609 30785308 PMC6292368

[225] Mathieu M. Martin-Jaular L. Lavieu G. Théry C.. Specificities of secretion and uptake of exosomes and other extracellular vesicles for cell-to-cell communication. Nature Cell Biology 21 (2019) 9-17. https://doi.org/10.1038/s41556-018-0250-9. 10.1038/s41556-018-0250-9 30602770

[226] Herrmann I.K. Wood M.J.A. Fuhrmann G.. Extracellular vesicles as a next generation drug delivery platform. Nature Nanotechnology 16 (2021) 748-759. https://doi.org/10.1016/j.biopha.2022.113480. 10.1016/j.biopha.2022.113480 34211166

[227] Haney M.J. Klyachko N.L. Zhao Y. Gupta R. Plotnikova E.G. He Z. Patel T. Piroyan A. Sokolsky M. Kabanov A.V. Batrakova E.V.. Exosomes as drug delivery vehicles for Parkinson’s disease therapy. Journal of Controlled Release 207 (2015) 18-30. https://doi.org/10.1016/j.jconrel.2015.03.033. 10.1016/j.jconrel.2015.03.033 25836593 PMC4430381

[228] Kim M.S. Haney M.J. Zhao Y. Mahajan V. Deygen I. Klyachko N.L. Inskoe E. Piroyan A. Sokolsky M. Okolie O. Hingtgen S.D. Kabanov A.V. Batrakova E.V.. Development of exosome-encapsulated paclitaxel to overcome MDR in cancer cells. Nanomedicine: Nanotechnology, Biology and Medicine 12 (2016) 655-664. https://doi.org/10.1016/j.nano.2015.10.012. 10.1016/j.nano.2015.10.012 26586551 PMC4809755

[229] Feng T. Tang Z. Karges J. Shen J. Jin C. Chen Y. Pan Y. He Y. Ji L. Chao H.. Exosome camouflaged coordination-assembled Iridium(III) photosensitizers for apoptosis-autophagy-ferroptosis induced combination therapy against melanoma. Biomaterials 301 (2023) 122212. https://doi.org/10.1016/j.biomaterials.2023.122212. 10.1016/j.biomaterials.2023.122212 37385136

[230] Ibraheem D. Elaissari A. Fessi H.. Gene therapy and DNA delivery systems. International Journal of Pharmaceutics 459 (2014) 70-83. https://doi.org/10.1016/j.ijpharm.2013.11.041. 10.1016/j.ijpharm.2013.11.041 24286924

[231] Danaeifar M.. Recent advances in gene therapy: genetic bullets to the root of the problem. Clinical and Experimental Medicine 23 (2023) 1107-1121. https://doi.org/10.1007/s10238-022-00925-x. 10.1007/s10238-022-00925-x 36284069

[232] Wang Y. Ji X. Ruan M. Song R. Dai J. Xue W.. Worm-like biomimetic nanoerythrocyte carrying siRNA for melanoma gene therapy. Small 14 (2018) 1803002. https://doi.org/10.1002/smll.201803002. 10.1002/smll.201803002 30334353

[233] Han H.J. Ekweremadu C. Patel N.. Advanced drug delivery system with nanomaterials for personalised medicine to treat breast cancer. Journal of Drug Delivery Science and Technology 52 (2019) 1051-1060. https://doi.org/10.1016/j.jddst.2019.05.024. 10.1016/j.jddst.2019.05.024

[234] Fornaguera C. García-Celma M.. Personalized nanomedicine: a revolution at the nanoscale. Journal of Personalized Medicine 7 (2017) 12. https://doi.org/10.3390/jpm7040012. 10.3390/jpm7040012 29023366 PMC5748624

